# Gold Nanoparticle Formation via X-ray Radiolysis Investigated with Time-Resolved X-ray Liquidography

**DOI:** 10.3390/ijms21197125

**Published:** 2020-09-27

**Authors:** Hosung Ki, Sungjun Park, Seunghwan Eom, Jain Gu, Siin Kim, Changwon Kim, Chi Woo Ahn, Minseo Choi, Sena Ahn, Doo-Sik Ahn, Jungkweon Choi, Mu-Hyun Baik, Hyotcherl Ihee

**Affiliations:** 1 Department of Chemistry, Korea Advanced Institute of Science and Technology (KAIST), Daejeon 34141, Korea; kihosung@kaist.ac.kr (H.K.); mnb0707@gmail.com (S.P.); eomshwan@gmail.com (S.E.); gujain33@gmail.com (J.G.); siin0308@gmail.com (S.K.); cwkim456@gmail.com (C.K.); acw116@kaist.ac.kr (C.W.A.); atlady05@gmail.com (M.C.); senatureahn@gmail.com (S.A.); doosikahn@gmail.com (D.-S.A.); cjkbabo@gmail.com (J.C.); mookiebaik2805@gmail.com (M.-H.B.); 2 KI for the BioCentury, Korea Advanced Institute of Science and Technology (KAIST), Daejeon 34141, Korea; 3 Center for Nanomaterials and Chemical Reactions, Institute for Basic Science (IBS), Daejeon 34141, Korea; 4 Center for Catalytic Hydrocarbon Functionalizations, Institute for Basic Science (IBS), Daejeon 34141, Korea

**Keywords:** X-ray radiolysis, gold nanoparticle, time-resolved X-ray liquidography, laser ablation

## Abstract

We report the generation of gold nanoparticles (AuNPs) from the aqueous solution of chloro(2,2′,2″-terpyridine)gold(III) ion ([Au(tpy)Cl]^2+^) through X-ray radiolysis and optical excitation at a synchrotron. The original purpose of the experiment was to investigate the photoinduced structural changes of [Au(tpy)Cl]^2+^ upon 400 nm excitation using time-resolved X-ray liquidography (TRXL). Initially, the TRXL data did not show any signal that would suggest structural changes of the solute molecule, but after an induction time, the TRXL data started to show sharp peaks and valleys. In the early phase, AuNPs with two types of morphology, dendrites, and spheres, were formed by the reducing action of hydrated electrons generated by the X-ray radiolysis of water, thereby allowing the detection of TRXL data due to the laser-induced lattice expansion and relaxation of AuNPs. Along with the lattice expansion, the dendritic and spherical AuNPs were transformed into smaller, raspberry-shaped AuNPs of a relatively uniform size via ablation by the optical femtosecond laser pulse used for the TRXL experiment. Density functional theory calculations confirm that the reduction potential of the metal complex relative to the hydration potential of X-ray-generated electrons determines the facile AuNP formation observed for [Au(tpy)Cl]^2+^.

## 1. Introduction

Information on the structural dynamics of a chemical reaction is crucial for determining its reaction mechanism. Time-resolved X-ray liquidograpy (TRXL), known as time-resolved X-ray solution scattering, is an established technique in this regard, by which the structural dynamics of molecules spanning diatomic molecules to proteins have been successfully investigated [1,2,3,4,5,6,7,8,9,10,11,12,13,14,15,16,17]. In a typical TRXL experiment [16,17,18], with X-ray facilities such as synchrotrons and X-ray free-electron lasers (XFELs), a liquid solution containing the solute molecule of interest is exposed to a femtosecond laser pulse to initiate a photochemical reaction of the solute molecule, and the structural changes associated with the subsequent reactions of the solute molecule are probed by an X-ray scattering of the solution sample as a function of time delays between the laser pulse and the X-ray pulse. In most TRXL experiments [1,2,3,4,5,6,7,8,9,10,11,12,13,14,15,16,17,18], X-rays serve well as a means of probing the reaction, but in some rare cases, the X-ray itself induces unwanted responses. Especially when the solute molecule contains metal atoms, they have the potential to be reduced by the stimulation of the X-ray and eventually form nanoparticles. So far, the characteristics of molecules responsible for determining whether the molecule would undergo such reduction and nanoparticle formation have been overlooked. In our effort to investigate the structural dynamics of chloro(2,2′,2″-terpyridine)gold(III) ion ([Au(tpy)Cl]^2+^, tpy = 2,2′:6′,2″-terpyridine) in water, we accidentally observed that the [Au(tpy)Cl]^2+^ undergoes a nanoparticle formation—in this case, the formation of gold nanoparticles (AuNPs). This observation provides useful information for studying nanoparticle formation induced by X-ray radiolysis and exploring the determining factor for the X-ray-induced generation of nanoparticles.

AuNPs have a wide range of applications in various fields such as catalysis, photonics, and biotechnology due to their unique size- and shape-dependent properties [19,20,21,22,23,24,25,26]. There are multiple methods for synthesizing AuNPs. The oldest and most representative synthetic method is chemical synthesis such as the Brust–Schiffrin method [27] and Turkevich method [28] using chloroauric acid as reaction precursors and sodium borohydride or citric acid as a reducing agent in the solution. Another technique involves electrochemical synthesis that reduces the precursor electrochemically at the electrode. Recently, a way of synthesizing AuNPs using pulse radiolysis has also been explored [29,30,31,32,33,34,35]. In this method, AuNPs are synthesized by the reduction of HAuCl_4_ via solvated electrons generated by gamma rays, X-rays, or intense optical to near-infrared femtosecond pulses. This method has relevance with our observation of AuNPs formation in the TRXL experiment. To study the optical properties of AuNPs generated over time, UV–visible extinction spectra were measured, and the characteristics of the size and shape of the generated AuNPs were checked with transmission electron microscopy (TEM) and powder X-ray diffraction (PXRD). Following the optical excitation by a laser pulse, the shape and angular shift of the Bragg reflections from crystalline AuNPs are resolved stroboscopically using 100 ps X-ray pulses from a synchrotron. These observations indicate lattice expansion and the subsequent relaxation of AuNPs excited by the 400-nm femtosecond laser pulse, which is used as the pump for the pump-probe TRXL experiment.

In our study, we used [Au(tpy)Cl]Cl_2_ as a metal source, whereas HAuCl_4_ was used in most of the studies reporting the nanoparticle formation by the pulse radiolysis. In contrast, no formation of nanoparticles was observed in TRXL experiments on the aqueous solutions of K[Au(CN)_2_] [7,9], K_4_[Pt_2_(P_2_O_5_H_2_)_4_] [4] and [Co(tpy)_2_](PF_6_)_2_ [2]. The observed differences in the generation of nanoparticles depending on the metal precursor suggest that the reduction potential (RP) of the cationic metal center to a neutral metal atom, which is determined by the oxidation number of the metal and its surrounding ligands, relative to the hydration potential (HP) of electron, decides the formation of nanoparticles. Indeed, the RPs of all metal-containing molecules in water studied by TRXL as well as HAuCl_4_ and [Au(tpy)Cl]Cl_2_ confirm this conclusion.

## 2. Results and Discussion

### 2.1. Formation of AuNPs Induced by X-ray Radiolysis

**Figure 1:**
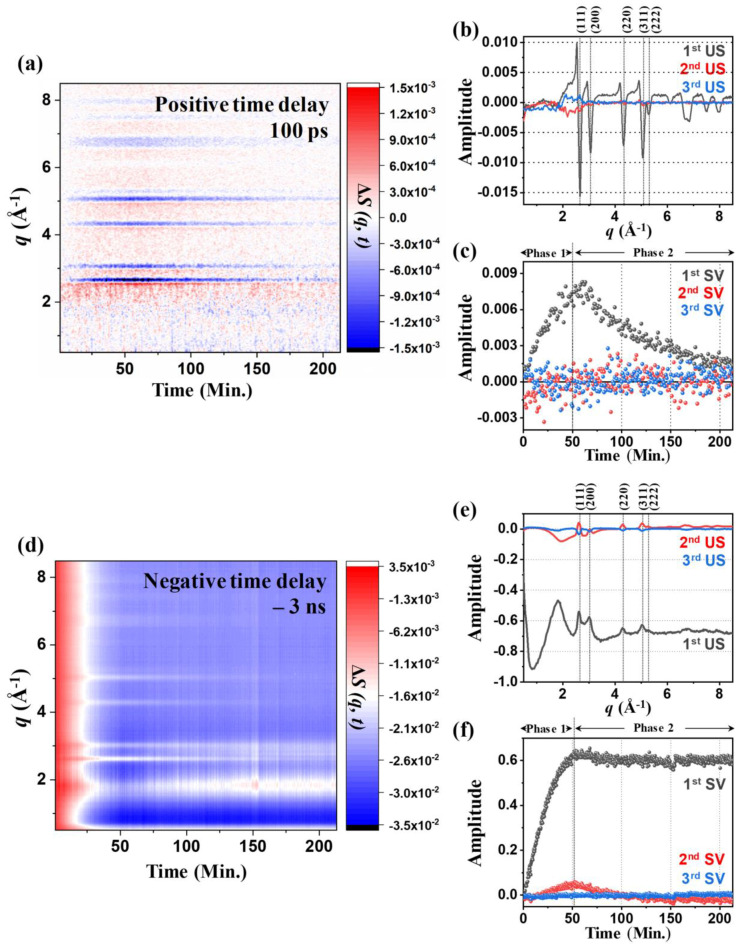
(**a**) Time-resolved X-ray liquidograpy (TRXL) difference curves at the time delay of 100 ps as a function of data collection time (*t*) ranging from 0 to 213 min. (**b**) The right upper panel shows the first three significant left singular vectors (lSVs) obtained from the singular value decomposition (SVD) analysis of (**a**). The peaks corresponding to the different lattice planes in face-centered cubic (fcc) gold are labeled. (**c**) The first three significant right singular vectors multiplied by singular values are shown. (**d**) Static difference X-ray scattering (SDXS) curves generated by subtracting the scattering curve measured on the fresh sample before the experiment from those during the experiment. The time delay (*t*) ranges from 0 to 213 min. (**e**) The upper panel shows the first three lSVs obtained from the SVD analysis of (**d**). The peaks corresponding to the different lattice planes in fcc gold are labeled. (**f**) The first three right singular vectors multiplied by singular values are shown.

The original purpose of the TRXL experiment on the [Au(tpy)Cl]Cl_2_ aqueous solution was to investigate the structural dynamics of [Au(tpy)Cl]^2+^. For example, in previous TRXL experiments on [Au(CN)_2_^−^]_3_ in water [7,9], the bond formation dynamics of a gold trimer complex was unveiled. We expected that analogous bond forming events may afford oligomers and intended to study its associated structural dynamics. In a typical TRXL experiment, scattering data are obtained at intervals of tens of time delays spanning a wide range from 0 to 3 μs. In addition, the scattering data at a negative time delay (for example, −3 ns) are also collected as a reference. Time-resolved difference data are obtained by subtracting the reference scattering data from the scattering data at each time delay. The scattering data are usually collected in the order of time delays. This cycle of data collection is repeated many times and averaged to attain a sufficient signal-to-noise ratio. Surprisingly, the TRXL experiments did not reveal any difference signals assignable to the structural changes of the solute molecules initially. However, after ~5 min, the difference curves suddenly started to show sharp peaks and valleys. As an example, the difference curves at the time delay of 100 ps as a function of data collection time are shown in Figure 1a.

### 2.2. Kinetics of the 100 PS TRXL Data

To obtain more detailed information on the kinetics of the reaction, we applied a singular value decomposition (SVD) to the data shown in Figure 1a. The right singular vectors (rSVs) and left singular vectors (lSVs) show that a single component, which is the first vector, alone can account for the observed data (Figure 1b,c). The intensities of the difference curves at 100 ps increase up to 50 min (phase 1) and then decrease in the subsequent 163 min (phase 2). At the same time, as shown in Figure S1, the color of the solution, which had been transparent yellow before the experiment, turned into dark purple as the experiment proceeded. The sudden appearance of difference scattering signals and the color change of the solution together indicated that the [Au(tpy)Cl]Cl_2_ aqueous solution was transformed into a different solution and the newly formed species must be responsible for the dynamics observed in the TRXL data.

We also measured the UV–visible spectra of the sample before, during, and after the TRXL experiment. For this UV–visible extinction measurement, we performed a TRXL experiment with a slight modification of the scheme for the supply of the sample. In the modified scheme, the sample was not continuously circulated but flowed from the reservoir bottle to the receiver bottle. By doing so, the sample, after passing the sample position of X-ray and laser pulses, was collected in the receiver bottle. Then, an aliquot of the sample in the receiver bottle was taken for a UV–visible extinction measurement and the remaining sample was moved to the reservoir bottle to repeat another round of passage from the reservoir bottle to the receiver bottle, from which an aliquot was taken for a UV–visible extinction measurement. Using this experimental scheme, we quantitatively monitored the spectral change of the sample as a function of the number of passages to X-ray and laser pulses. The samples after up to eight passages were collected, and the UV–visible spectra corresponding to the collected solutions are shown in Figure S2. The spectra show that the shoulder and peaks at 290, 350, and 366 nm characteristic of [Au(tpy)Cl]^2+^ quickly diminish and eventually disappear. Concomitantly, new peaks at 320 and 550 nm grow with time. As the sample solution is repeatedly exposed to the laser and X-ray pulses, the initial valley at ~440 nm moves toward a shorter wavelength. Accordingly, the extinction at 400 nm, which is the wavelength of the used femtosecond laser pulses, diminishes over time.

Sharp positive and negative peaks observed at specific *q* values in Figure 1a are characteristic of crystalline samples, and the peak positions turned out to be the same as those for typical gold crystals in the face-centered cubic (fcc) phase [36]. These observations indicate that the formation of gold crystals are responsible for the sharp peaks in the TRXL data. To further confirm this, we took the solution sample after the TRXL experiment and centrifuged it to separate the solid product from the supernatant. Both the solid product and the supernatant were further dried for PXRD measurements, and their PXRD patterns were compared with that of the initial sample ([Au(tpy)Cl]Cl_2_) as shown in Figure S3. The PXRD pattern from the solid product is indeed the same as that for the fcc gold crystals. The PXRD pattern from the supernatant is the same as that of the initial sample except for minor contaminant peaks due to the gold crystal that could not be completely removed by the centrifuge.

### 2.3. Kinetics of Static Difference X-ray Scattering (SDXS) Data and Comparison with the Kinetics of TRXL Data

To further investigate the time scale for the formation of the gold crystals, we also checked how the scattering curves measured at the negative reference time delay (−3 ns) change over the data collection cycles by subtracting the scattering curve of the first cycle from those measured for the following data collection cycles as shown in Figure 1d. Such static differences in X-ray scattering (SDXS) data contain information on the interatomic arrangement of the gold crystals. Thus, the kinetics information on the slow time scale can be deduced by tracking the time profile of the SDXS data.

To extract kinetic information, we conducted SVD on the SDXS data. The SVD result shows that two components are needed to explain the SDXS data (Figure 1e). During the initial 50 min (phase 1) of the experiment, both the first and the second rSV of SDXS data significantly rise. Note that this kinetic behavior is consistent with that of the first rSV of TRXL data during phase 1. This correlation again confirms that the formation of gold crystals is responsible for the rise in the intensity of the peaks in the TRXL signal during phase 1. In contrast, during the subsequent 150 min (phase 2), TRXL and SDXS data show noticeably different behavior. While the amplitude of TRXL data significantly decreases during phase 2, that of SDXS data remains almost unchanged. This inconsistent behavior of TRXL and SDXS data indicates that the reason for the decrease in TRXL data during phase 2 is not the opposite of the process that occurs in phase 1.

During phase 2, only the shape of the SDXS data changes, further indicating that the change of the shape of SDXS would be closely related to the decrease in TRXL data. The first lSV displays the representative, overall shape of all SDXS curves (Figure 1e). One of the representative features of the first lSV is the positive peaks, which are positioned at around *q* = 2.6, 3.0, 4.3, and 5.1 Å^−1^. The positions of the positive peaks are consistent with those of the fcc gold crystals, indicating that the formation of the gold crystals is responsible for the evolution of SDXS data. Another representative feature of the first lSV is the negative baseline over the entire *q*-range, which indicates that the electron density of the sample solution decreases during the experiment. Along with the formation of the gold crystals, the concentration of the [Au(tpy)Cl]^2+^ in the solution would decrease because [Au(tpy)Cl]^2+^ is the only source of Au atoms necessary for the growth of crystalline gold. In addition, a certain fraction of the gold crystals formed in the solution would precipitate rather than being dispersed in solution, resulting in the further decrease in electron density of the sample solution. The presence of the second significant lSV indicates that, as shown in Figure 1e, the shape of SDXS curves changes over time during the experiment. Representatively, Figure 1d shows that the amplitude of the positive peak at around *q* = 1.9 Å^−1^ generally increases, and those of other positive peaks at around *q* = 2.6, 3.0, 4.3, and 5.1 Å^−1^ decrease with time. A reduction in the size or the crystallinity of the initially (within ~50 min from the beginning of the TRXL experiment) formed gold crystals can be a potential candidate to explain the amplitude decrease in the peaks which correspond to the fcc gold crystals.

### 2.4. TEM Images and Formation Mechanism of AuNPs

**Figure 2:**
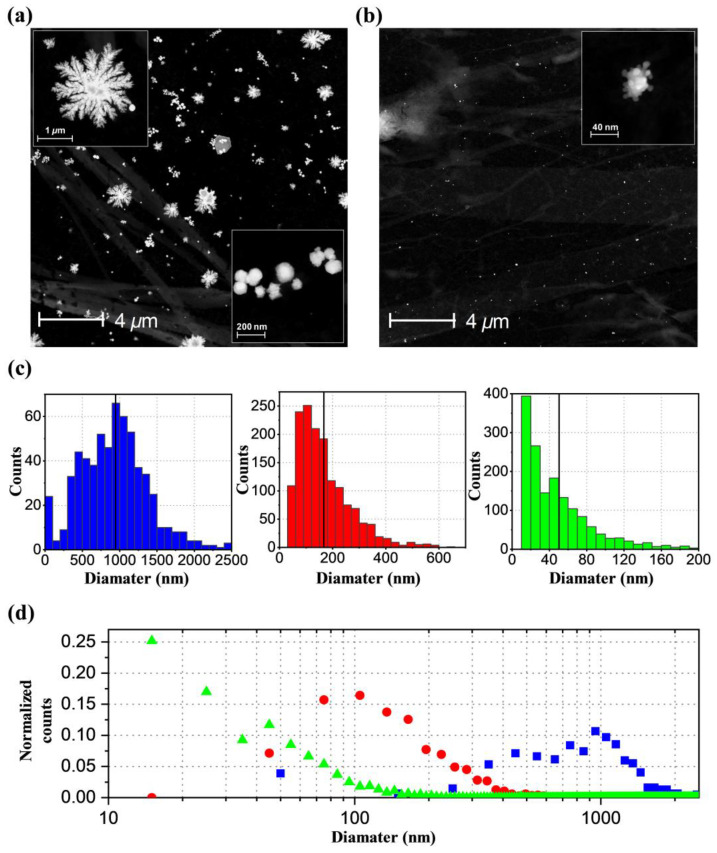
(**a**) TEM image showing the overall distribution of gold nanoparticles (AuNPs) in phase 1. The insets show enlarged views for dendritic (left upper) and spherical (right bottom) AuNPs. (**b**) TEM image showing the overall distribution of AuNPs in phase 2. The inset shows an enlarged view of raspberry-shaped AuNPs. A further magnified image is shown in Figure S5. (**c**) Size histograms of the dendritic- (blue), spherical- (red), and raspberry-shaped (green) AuNPs. Note that the y-axis scale is different for each histogram. The black solid line in each panel represents the average size. All images used to construct histograms are shown in Figures S4 and S6. (**d**) Comparison of the size distribution of the dendritic- (blue), spherical- (red), and raspberry-shaped (green) AuNPs. The counts in each histogram shown in (**c**) are normalized by dividing by the sum of the counts in all bins. The resulting normalized counts for the dendritic-, spherical-, and raspberry-shaped AuNPs are shown together for the comparison. The solid lines represent the average size. Note that the x-axis is on the logarithmic scale.

To gain more detailed insights into what happens during phase 1 and phase 2, we sampled the sample solution at phase 1 and phase 2 and further investigated the sample solutions by collecting TEM images. Figure 2a and Figure S4 show TEM images of representative AuNPs in phase 1. There are two types of shapes, dendrites, and spheres. The dendritic AuNPs have an average size of ~950 nm and a standard deviation of 500 nm, and the spherical AuNPs are 170 ± 100 nm.

**Figure 3:**
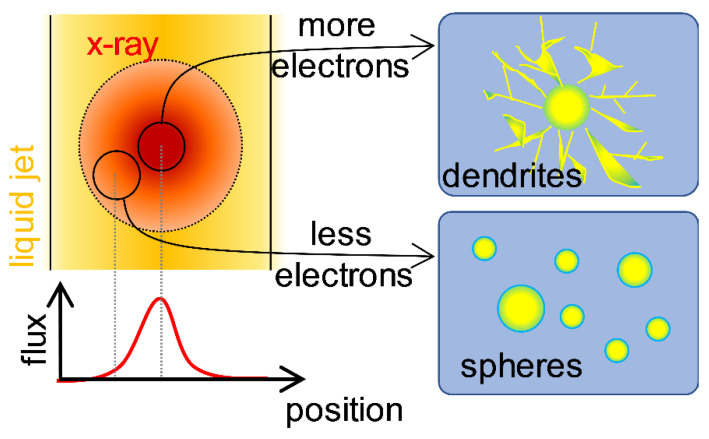
A schematic diagram showing the origin of the formation of two different types of AuNPs by X-ray radiolysis. The X-ray pulse used in this study has a Gaussian-shaped spatial distribution of intensity. At the central region of the X-ray pulse, where the intensity of the X-ray is higher, a higher number of hydrated electrons are formed, and the reduction of the Au complex is much faster. Accordingly, the AuNPs grow in a kinetically favored fashion. In contrast, at the boundary of the X-ray pulse, a smaller number of hydrated electrons are formed, the reduction of the Au complex is much slower, and thus the AuNPs grow in a thermodynamically favored way.

The formation of two types of morphology indicates that two different formation mechanisms may be operational. The AuNP formation requires the reduction of gold(III) so that the resulting gold(0) atoms can nucleate and grow into AuNPs. The most plausible candidate for the reducing agent is the hydrated electron, which can be generated by the X-ray radiolysis of water [29,30,31,32,33,34,35]. It is known that a low concentration of hydrated electrons results in the reaction-limited growth, which generates 3D faceted or sphere-shaped NPs, whereas a high concentration of hydrated electrons results in the diffusion-limited growth to afford NPs with dendritic morphologies [37]. In addition, it has been reported that the shape of Au and silver NPs changes depending on the dose rate when preparing NPs using gamma ray radiolysis [33,38,39]. Therefore, the appearance of two types of morphologies can be rationalized by considering the off-center and center regions of the X-ray pulse that can generate hydrated electrons of lower and higher concentrations, respectively. These electrons promote two different reduction mechanisms, resulting either in reaction-limited or diffusion-limited growths (See Figure 3). Further calculations support the feasibility of the hypothesis that the two different types of AuNPs can be formed during phase 1 via different growth mechanisms (see Figure S7, and “Simulation of photoelectron generation in liquid jet subjected to X-ray pulse” section in the Supplementary Materials).

To take a closer look at what happened during phase 1, especially regarding the evolution of the size and shape of the AuNPs upon repeated exposure to X-ray and laser pulses, we performed additional TEM measurements on samples collected as a function of the number of passages to X-ray and laser pulses, as we did for the UV–visible extinction measurements, shown in Figure S2. Samples were collected after up to eight passages, and the TEM images of the AuNPs present in each solution were taken. The measured TEM images confirm that the shape of the AuNPs is consistent in all the sample solutions having different amounts of exposure to X-ray and laser pulses: two types of particles, dendritic and spherical AuNPs, were observed. The particle size of each of the dendritic and spherical AuNPs and the relative ratio of the number of dendritic AuNPs to spherical AuNPs do not show strong dependence on the amount of exposure to X-ray and laser pulses (See Figure S8). This result is also consistent with the UV–Vis spectra shown in Figure S2, which do not show any noticeable change of the shape and peak positions.

The TEM images for the sample taken 100 min after the experiment had been performed (phase 2) show that AuNPs have raspberry-like shapes, unlike the dendrite or spherical shapes observed in phase 1 (See Figures S5 and S6). The raspberry-like particles have an average size of ~50 nm and a standard deviation of 44 nm. This observation indicates that, regardless of the initial size and shape of the AuNPs, the initially formed AuNPs are all converted to raspberry-shaped AuNPs by the irradiation of femtosecond laser pulses used for the TRXL experiment. In previous studies on AuNP formation by X-ray [29,30,33,34,35,40] or optical pulses [41,42,43,44,45,46], the morphologies of the generated AuNPs were mostly spheres, polyhedrons, and rods. Our case is unique in that both the formation of AuNPs and its laser-induced structural dynamics were simultaneously studied in a single experiment, whereas, in previous studies, either of the two techniques was employed separately. The unique combination of both X-ray and optical pulses allowed for the generation of various types of AuNPs and their time-dependent conversion. The TEM images indicate that AuNPs exist throughout both phase 1 and phase 2, although the shape and size distribution of AuNPs change. Thus, the resulting solution contained raspberry-shaped AuNPs even when the TRXL signal stopped showing any distinct signal, which explains why the SDXS data show signals due to nanoparticles even toward the end of phase 2. This finding raises the question of why the TRXL signal decreases with time and eventually stops showing any signal toward the end of phase 2. This question can be answered by considering that the UV–visible spectrum of the final solution containing raspberry-shaped AuNPs shows the spectral valley at 400 nm and thus has almost zero extinction at that wavelength (Figure S2). In other words, the raspberry-shaped AuNPs, which are the final products, only have negligible extinction at the wavelength of the femtosecond laser pulse, and thus no further excitation occurs. Since there is no photoinduced reaction anymore, the TRXL data do not have any photoinduced signal.

### 2.5. Lattice Dynamics and Ablation of AuNPs

**Figure 4:**
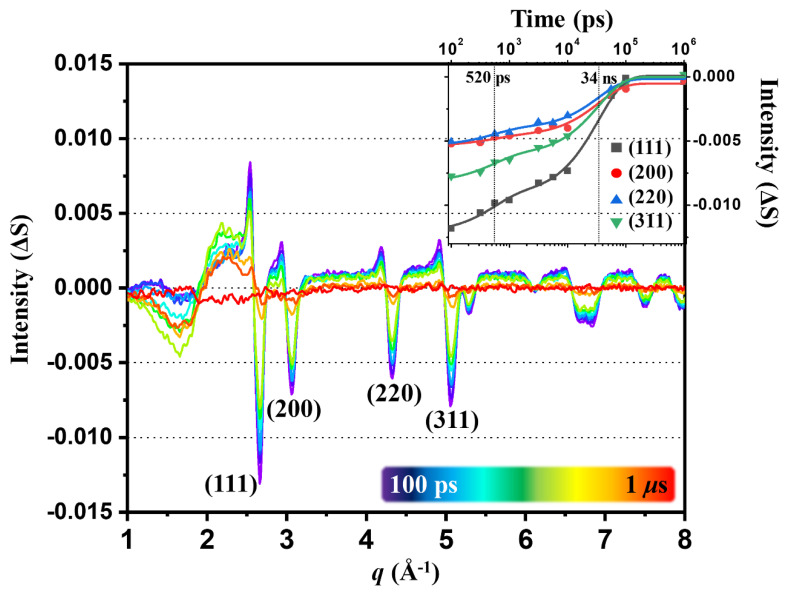
TRXL curves measured at time delays from 100 ps to 1 μs for the in situ-generated gold nanoparticles in solution. The significant peaks corresponding to the different lattice planes in fcc gold are labeled. The inset shows the time profile of the Bragg scattering intensities of the peaks corresponding to the reflection planes of fcc gold crystal.

The time-resolved difference curves at other time delays from 100 ps to 1 μs, such as those shown for 100 ps in Figure 1a, were also analyzed in the same manner with SVD and the first lSV components as a function of time are shown in Figure 4. The intensities of the positive and negative peaks of TRXL data display their maximum values at 100 ps and decrease with time and at 1 μs, the peaks completely disappear. Generally, each of the strong negative peaks is coupled with a positive peak at slightly smaller *q*, indicating the lattice is expanded. The positions of negative peaks are consistent with those expected for typical fcc gold crystals. Previously, these features of coupled positive and negative peaks assignable to lattice expansion were observed in TRXL experiments where spherical AuNPs of a well-defined size suspended in water were used [36]. To extract the kinetics of this laser-induced process, we plotted the intensities of the major negative peaks as a function of time and fitted them with a sum of exponential decay functions sharing common time constants. A satisfactory fit was obtained with two time constants of 520 ± 110 ps and 34 ± 3 ns. The kinetics of spherical AuNPs with a narrow size distribution studied by TRXL showed a single kinetic component for the relaxation of transiently lattice-expanded nanoparticles. Time constants for various sizes of particles showed that the time constant increases with the average size [47,48]. Accordingly, the two kinetic components observed here can be attributed to the relaxation of two different types of particles, dendritic and spherical particles, which have considerably different sizes. Specifically, 520 ps and 34 ns correspond to the relaxation of transiently lattice-expanded spherical and dendritic nanoparticles, respectively. We note that in previous TRXL studies on AuNPs, the morphologies of AuNPs were not characterized by TEM, leaving uncertainty regarding the morphology change induced by laser excitation in those studies. The oscillatory feature in 1~3 Å^−1^, which exhibits a completely different kinetic behavior, is due to the water heating signal.

It is known that the laser excitation of nanoparticles results in a significant rise in the local temperature. If the flux of the excitation laser is sufficiently high, the temperature of the particles can reach even the melting point of the particles, leading to the complete melting of the particles [47,49,50]. Such melting of the particles results in a severe reduction in the size and the change of the shape of the particles. It was previously reported that such complete melting and reduction in size of the particles could be monitored by tracking the intensity of Bragg peaks in the TRXL data. Upon the melting of the particles, the Bragg peaks completely disappear. Due to the subsequent reduction in the size of the particles after the melting, the intensities of the Bragg peaks do not completely recover even after the recrystallization of the particles. In contrast, if the flux of the laser is not high enough to melt the particles, only a shift of the positions of the Bragg peaks, which is attributed to the lattice expansion, is observed [47,51]. After the subsequent relaxation process, the position and amplitude of the peaks are completely recovered. Our TRXL data are consistent with the latter case, indicating that the laser flux in this experiment was not high enough to completely melt the particles.

Our SDXS data and TEM images show that the size of the particles is notably reduced, and their shape is also altered during phase 2 of the experiment. The notable point is that such a substantial change of the particles can occur without the melting of the particles, as the TRXL data indicate. In fact, previous studies reported that such reshaping [50] or size reduction [49,50] could occur even at a temperature far below the melting point. We suggest that surface ablation due to field enhancement on the particles is responsible for the observed reduction in the particle size [49,50]. Moreover, the TEM images indicate that the laser ablation in this experiment yields only the raspberry-shaped AuNPs regardless of the initial size and shape of the AuNPs, as mentioned above. This observation is consistent with a study reporting that the size of the nanoparticles after the laser ablation is almost independent of the initial size and shape of the nanoparticles [52].

### 2.6. Overall Picture

**Figure 5:**
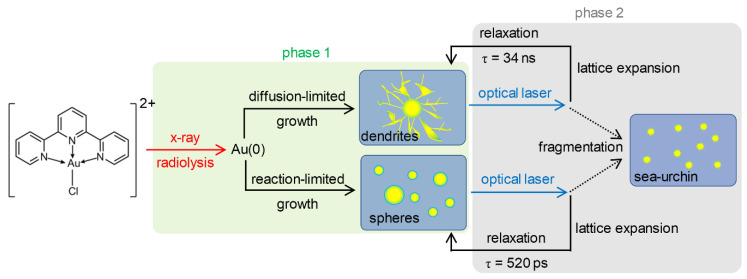
TRXL curves measured at time delays from 100 ps to 1 μs for the in situ-generated gold nanoparticles in solution. The significant peaks corresponding to the different lattice planes in fcc gold are labeled. The inset shows the time profile of the Bragg scattering intensities of the peaks corresponding to the reflection planes of fcc gold crystal.

The results of TRXL data, TEM measurements, UV–visible spectra, and PXRD data indicate two crucial aspects of the X-ray-induced formation and laser-induced lattice expansion and subsequent relaxation process of the AuNPs. First, two different types of nanoparticles with different morphologies and sizes are formed initially and eventually transform into a single type of much smaller nanoparticles with a uniform size. Second, the produced AuNPs are raspberry-shaped. These observations are consistent with the scenario depicted in Figure 5. Both the dendritic and spherical AuNPs formed by X-ray radiolysis transiently undergo lattice expansion by femtosecond laser pulses and subsequent relaxation, giving the observed TRXL signal. Through this process, a certain portion of AuNPs undergo fragmentation, making the nanoparticle size smaller, and resulting in raspberry-shaped AuNPs. We also checked the possibility that the optical laser pulses instead of X-ray pulses may be responsible for the AuNPs formation in a separate experiment where only femtosecond laser pulses at 400 nm without X-ray pulses were irradiated to the aqueous solution of [Au(tpy)Cl]Cl_2_. No AuNP formation was detected in this case, confirming that X-ray is responsible for the formation of AuNPs.

### 2.7. Determining Factor for Nanoparticle Formation by X-ray Radiolysis

We note that the formation of AuNPs was not observed in our TRXL experiment on K[Au(CN)_2_] in water, which was performed under similar experimental conditions [7,9]. Since the formation of nanoparticles involves the reduction of the metal and the reduction force is driven by the hydrated electrons generated by X-ray radiolysis, we theoretically evaluated the standard reduction potentials (E°) for the generation of the neutral metal atoms from AuCl_4_^−^, [Au(tpy)Cl]^2+^, and Au(CN)_2_^−^ in water using density functional theory (DFT) calculations and compared them to the hydration potential (HP) of an electron (E°(e^−^_gas_/e^−^_aq_)), which was determined experimentally to be −2.71 V [53] and computed to be −2.64 V [53]. The calculated E° values immediately show a strong correlation with the tendency to form nanoparticles (See Table S1 for the E° values, and Tables S2–S4 for the optimized geometries, vibrational frequencies, and detailed energy components obtained from the DFT calculations). For example, AuCl_4_^−^ and [Au(tpy)Cl]^2+^ in water, which form nanoparticles, have calculated E° values of 0.44 and 0.07 V, respectively, notably more positive than E°(e^−^_gas_/e^−^_aq_), whereas Au(CN)_2_^−^ in water, which does not form nanoparticles, has a calculated E° of–3.31 V, much more negative than E°(e^−^_gas_/e^−^_aq_). Then, we expanded the scope to all metal-bearing complexes in water which have been studied previously using TRXL, such as [Pt_2_(P_2_O_5_H_2_)_4_]^4–^ and [Co(tpy)_2_]^2+^ (Table S1) to obtain a clue for the relationship between the nanoparticle formation and the type of reactant molecule [2,4]. The calculated E° in water were −4.95 and −3.13 V, again being more negative than E°(e^−^_gas_/e^−^_aq_) and consistent with the trend. Thus, the trend found from AuCl_4_^−^, [Au(tpy)Cl]^2+^, and Au(CN)_2_^−^ appears to be general. All molecules successfully studied by TRXL without the undesirable nanoparticle formation have E° values that are more negative than E°(e^−^_gas_/e^−^_aq_), thus making the nanoparticle formation energetically unfavorable.

## 3. Materials and Methods

### 3.1. Preparation of [Au(tpy)Cl]Cl_2_

[Au(tpy)Cl]Cl_2_ was synthesized according to the reported method [54]. An equimolar amount of HAuCl_4_ (Samchun Chemical, Pyeongtaek, Korea) and terpyridine (Samchun Chemical, Pyeongtaek, Korea), which was refluxed in H_2_O at pH 3~5 for 24 hrs, gave the desired [Au(tpy)Cl]Cl_2_ in 80% yields along with traces of [Au(tpy)Cl]_2_[AuCl_2_]_3_[AuCl_4_] (Figure S9) [54]. The gold complex was mainly characterized by NMR spectroscopy, in which [Au(tpy)Cl]^2+^ showed downfield shifts for the 4,4″-tpy*H*s (Δδ = 0.51 ppm), 5,5″-tpy*H*s (Δδ = 0.46 ppm), and 6,6″-tpy*H*s (Δδ = 0.20 ppm) relative to the ligand due to the influence of the metal–ligand bond.

### 3.2. Time-Resolved X-ray Solution Scattering

**Figure 6:**
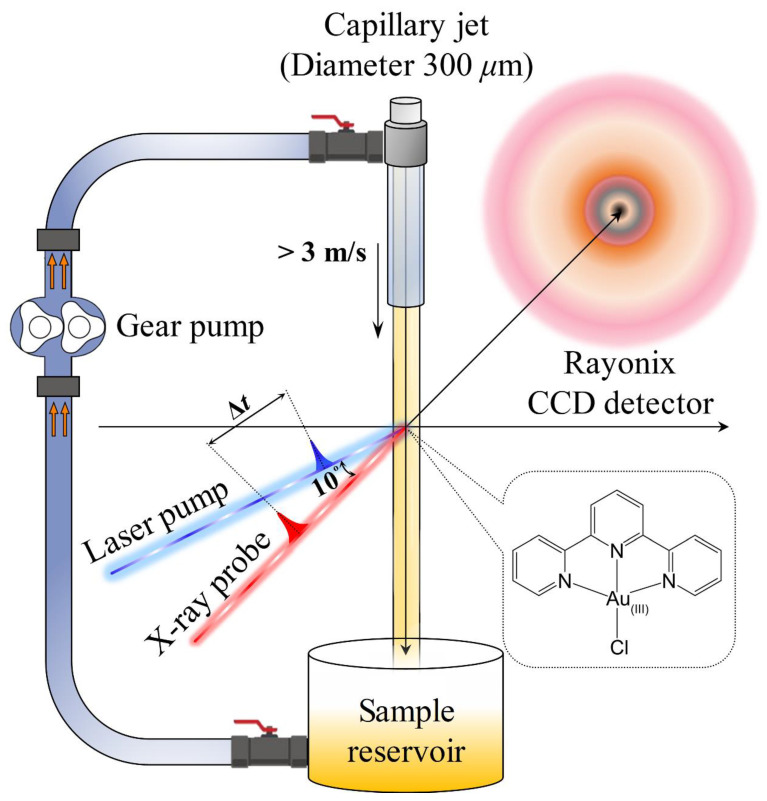
Scheme of the TRXL experiment on gold (III) terpyridine complex ([Au(tpy)Cl]Cl_2_) aqueous solution. The solution sample was circulated by a gear pump through the open jet sample delivery system, the sample reservoir, and the tubes. The solvated electrons generated by X-ray radiolysis of H_2_O induces the formation of gold nanoparticles, which do not exist early in the experiment. The generated AuNPs are pumped by a femtosecond laser pulse and, subsequently, a 100-ps X-ray pulse incident with a time delay, Δ*t*, probes lattice expansion and relaxation of AuNPs. The X-ray beam propagated along the *x*-axis, and the sample was flown along the *z*-axis.

The TRXL experiment was conducted at the ID09 beamline of the European Synchrotron Radiation Facility (ESRF). Details of the TRXL setup were reported elsewhere [5,16,17,18]. Briefly, the pump-probe scheme was applied to monitor the time-dependent X-ray scattering signals upon irradiation with an optical laser pulse (Figure 6). Laser pulses as a pump with the center wavelength of 400 nm were generated to initiate the photochemical reaction by the second-harmonic generation of the 800-nm femtosecond laser pulses provided by a 1-kHz-amplified Ti:sapphire laser system. The 400 nm laser pulses have an energy of 95 µJ/pulse and were focused on a 0.23 (width) × 0.29 (height) mm^2^ spot at the sample position, providing a fluence of ~1.8 mJ/mm^2^. After initiating the reaction by the laser pulse, a time-delayed X-ray pulse was delivered to the sample to probe the reaction dynamics. The ~100 ps-long X-ray pulses produced by an undulator were further monochromatized with a multilayer pair consisting of Ru/B_4_C installed at the beamline. The resulting X-ray pulses with a central energy of 18 keV and energy bandwidth of ΔE/E = ~0.9% were used for the TRXL experiment. The size of the X-ray pulses was 0.17 (width) × 0.11 (height) mm^2^ at the sample position. The scattered X-ray photons by the sample solution were collected by an area detector (Rayonix MX170-HS, 1920 × 1920 pixels, 89 mm pixel size) in the 2 × 2 binning mode with a sample-to-detector distance of 45 mm and an exposure time of three seconds per image. The sample solution of 10.0 mM [Au(tpy)Cl]Cl_2_ in distilled water was circulated through the open jet capillary system with a thickness of 500 μm. We obtained the solution scattering signals at the pump-probe time delays of *t* = −3 ns, 100 ps, 178 ps, 316 ps, 562 ps, 1 ns, 1.78 ns, 3.16 ns, 5.62 ns, 10 ns, 56.2 ns, 100 ns, and 1 μs. A total of 230 cycles were taken to collect the data. The difference scattering signals, ΔS(*q*, *t*), were obtained by subtracting the scattering signal from the unexcited sample, measured at a negative time delay (*t* = −3 ns), from the signals measured at the positive time delays.

## 4. Conclusions

Because the reduction potential E° of [Au(tpy)Cl]^2+^ is more positive than the hydration potential of the electron, the hydrated electrons generated by X-ray radiolysis effectively reduce [Au(tpy)Cl]^2+^ ion to form AuNPs. Different concentrations of hydrated electrons generate AuNPs in two different growth regimes, resulting in dendritic and spherical AuNPs with different size distributions. The generated AuNPs are excited by femtosecond laser pulses at 400 nm and undergo lattice expansion and relaxation, which give rise to visible TRXL signals. Via repeated laser ablation accompanying the lattice dynamics, dendritic and spherical AuNPs are transformed into smaller raspberry-shaped AuNPs of a uniform size. Raspberry-shaped AuNPs no longer absorb photons at 400 nm and thus stop giving visible TRXL signals.

## Supplementary Materials

The following are available online at https://www.mdpi.com/1422-0067/21/19/7125/s1, Figure S1: Comparison of sample states before and after the TRXL experiment, Figure S2: Evolution of UV-visible spectra of the sample solution upon exposures to the laser and x-ray pulses, Figure S3: PXRD patterns from the initial sample, the supernatant, and the solid product after the TRXL experiment, Figure S4: Six TEM images of the AuNPs in the sample solution collected in phase 1, Figure S5: A magnified TEM image of a raspberry-shaped AuNP, Figure S6: Six TEM images of the AuNPs in the sample solution which is collected at phase 2, Figure S7: Spatial distribution of the photoelectrons generated by an x-ray pulse, Figure S8: Evolution of the size and the number of dendritic and spherical AuNPs in the sample solution upon exposures to the laser and x-ray pulses, Figure S9: Synthesis of [Au(tpy)Cl]Cl_2_, Table S1: Hydration potential of an electron, standard reduction potential of the molecule studied in this work, and the molecules containing metal studied in previous TRXL work, and free energy changes of related reactions, Table S2: Cartesian coordinates of the optimized geometries, Table S3: Vibrational frequencies of the optimized structures, Table S4: Computed energy components for DFT-optimized geometries.

## Abbreviations

AuNPs	Gold nanoparticles
[Au(tpy)Cl]^2+^	Chloro(2,2′,2″-terpyridine)gold(III) ion
tpy	2,2′:6′,2″-Terpyridine
TRXL	Time-resolved X-ray liquidography
XFEL	X-ray free-electron laser
TEM	Transmission electron microscopy
PXRD	Powder X-ray diffraction
RP	Reduction potential
HP	Hydration potential
SVD	Singular value decomposition
rSV	Right singular vector
lSV	Left singular vector
fcc	Face-centered cubic
SDXS	Static difference X-ray scattering
E°	Standard reduction potentials
DFT	Density functional theory
ESRF	European Synchrotron Radiation Facility
