# Supplementary material for: Gold Nanoparticle Formation via X-ray Radiolysis Investigated with Time-Resolved X-ray Liquidography
Source: Int J Mol Sci. 2020 Sep 27;21(19):7125. doi: 10.3390/ijms21197125 (PMC7582564; doi:10.3390/ijms21197125)
Supplement: Supplementary file 1 [file ijms-21-07125-s001.zip › ijms-931329_proofread_si_annotated.pdf]

Supporting Information for

Gold nanoparticle formation via x-ray radiolysis investigated with time-resolved x-ray liquidography

Hosung Ki [1,2,3], Sungjun Park [1,2,3], Seunghwan Eom [1,2,3,4], Jain Gu [1,2,3], Siin Kim [1,2,3], Changwon Kim [1,2,3], Chi Woo Ahn [1,2,3], Minseo Choi [1,2,3], Sena Ahn [1], Doo-Sik Ahn [1,2,3], Jungkweon Choi [1,2,3], Mu-Hyun Baik [1,4], Hyotcherl Ihee *[,1,2,3]

*To whom correspondence should be addressed. E-mail: hyotcherl.ihee@kaist.ac.kr

This PDF file includes:

Supporting Methods S1-S3

Supporting Tables S1-S4

Supporting Figures S1-S9

Supporting References

Supporting Methods

Method S1. Experimental methods

S1.1. TEM measurement

During the TRXL experiment, aliquots of the sample in the reservoir bottle were taken at 5 minutes for phase 1 and 213 minutes for phase 2 after the experiment had been started. These aliquots were centrifuged to precipitate AuNPs. Supernatants of the solution were removed after centrifugation. The precipitates were re-dispersed in deionized water and dried over a TEM grid. To clearly visualize the structure of AuNPs over the backgrounds from organic impurities, high-angle annular dark field-scanning transmission electron microscopy (HAADF-STEM) images of the samples were obtained. The TEM measurements were performed using an FEI Titan G2 ETEM F30 microscope with an operating voltage of 300 kV.

S1.2. PXRD measurement

After the TRXL experiment, we collected an aliquot of the sample solution. The solution was centrifuged to separate the solid products in the solution as a precipitate. The resulting precipitate was sampled and dried in the air to measure powder x-ray diffraction (PXRD) patterns. The supernatant after the centrifugation was collected as well and dried by using a rotary evaporator at room temperature to measure the PXRD patterns of the remaining chemical components in the supernatant. The PXRD patterns of the samples were collected by a Rigaku SmartLab diffractometer using a nickel-filtered Cu Kα radiation beam (40 kV, 30 mA).

Method S2. Simulation of photoelectron generation in the liquid jet exposed to an x-ray pulse

S2.1. Spatial distribution of the intensity of the x-ray pulse

We assumed a Gaussian-shaped spatial distribution of intensity of x-ray pulse. Considering the FWHM size of the x-ray pulse at the sample position (170 (width) × 110 (height) μm^2), the intensity of the x-ray pulse at a position, (x, y), which is separated by x μm in horizontal direction and y μm in vertical direction from the center of the x-ray pulse was described using the following equation:

$$I_0(x,y) = c \cdot \exp(-x^2/[2.0\cdot(170/2\sqrt{2\ln 2})^2] - y^2/[2.0\cdot(110/2\sqrt{2\ln 2})^2]) \quad \text{(S1)}$$

where c is a constant.

S2.2. Absorption of x-ray and generation of photoelectrons

Based on the spatial distribution of the intensity of the x-ray pulse, $I_0(x, y)$, calculated by using Equation (S1), we estimated the number of photoelectrons generated by the x-ray pulse by using the following equations:

$$I(x,y) = I_0(x,y)\cdot\exp(-\mu_{\mathbf{m}}\cdot\rho\cdot\lambda) \quad \text{(S2)}$$

$$n_e(x,y) = I_{abs}(x,y) = I_0(x,y) - I(x,y)\cdot[1-\exp(\mu_m\cdot\rho\cdot\lambda)] \quad \text{(S3)}$$

$$\Phi_e(x,y) = n_e(x,y)/(\tau\cdot\lambda) \quad \text{(S4)}$$

where $I(x, y)$ is the intensity of the x-ray pulse after passing through the sample at (x, y), μ_m is the photoelectric absorption mass attenuation coefficient for the sample, ρ is the density of the sample, λ is the pass length of the x-ray pulse in the sample, $n_e(x, y)$ is the number of photoelectrons generated by the x-ray pulse at (x, y), $I_{abs}(x, y)$ is the number of x-ray photons absorbed for the generation of photoelectrons at (x, y), $\Phi_e(x, y)$ is the rate of photoelectron generation during the duration of x-ray pulse per unit volume at (x, y), and τ is the temporal duration of the x-ray pulse. We calculated $n_e(x, y)$ with the following experimental parameters: μ_m = 0.7636 cm^2/g [1], ρ = 0.997 g/cm^3, λ = 300 μm = 0.03 cm, and τ = 100 ps. Note that we assumed that μ_m and ρ for the sample solution are the same to those for the pure liquid water.

According to our calculation, about 2.28 percent of the incident x-ray photons are absorbed by the liquid jet to generate the photoelectrons. The resulting distribution of $\Phi_e(x, y)$ is depicted in Figure ~~S6~~ S7 in $e^-/(Å^3{\cdot}s)$ units.

S2.3. Comparison to a previous TEM study

As shown in Figures 3 and 5 of the main text, we suggest that two different types of AuNPs can be formed under our TRXL experimental condition because AuNPs can be produced via two different growth mechanisms, diffusion-limited and reaction-limited growth. To quantitatively support our suggestion, we compared the calculated rate of photoelectron generation by the x-ray pulse, $\Phi_e(x, y)$, with the experimental parameters reported from the previous study [2]. In the previous study, the formation of AuNPs by irradiation of electrons, not x-rays, to the aqueous solution of $HAuCl_4$ in water was investigated by varying the dose rate of electrons and the concentration of $HAuCl_4$. As a result, it was observed that spherical AuNPs are formed via reaction-limited growth at a low dose rate, and the dendritic AuNPs are formed via diffusion-limited growth at a high dose rate. We compared the reported dose rates for the diffusion-limited growth and the reaction-limited growth to $\Phi_e(x, y)$, as shown in Figure ~~S6~~S7. Specifically, it was reported that diffusion-limited growth of AuNPs was observed for the 5 mM concentration of $HAuCl_4$ at the dose rate of 5.30 $e^-/(Å^2{\cdot}s)$ and reaction-limited growth of AuNPs was observed at the dose rates of 1.4 and 2.74 $e^-/(Å^2{\cdot}s)$ for the 5 mM and 20 mM concentrations, respectively. We converted the dose rate values to the rate of electron absorption per unit volume by dividing by the thickness of the sample cell for the TEM experiment, 150 nm. The resulting values of the rate of electron absorption per unit volume are 3.53×10^{-3} $e^-/(Å^3{\cdot}s)$ (black dashed line) for the diffusion-limited growth of AuNPs for the 5 mM concentration, and 9.33×10^{-4} (yellow dashed line) and 1.83×10^{-3} $e^-/(Å^3{\cdot}s)$ (red dashed line) for the reaction-limited growth of AuNPs for the 5 mM and 20 mM concentrations,

respectively. By comparing the values to $\Phi_e(x, y)$, we confirmed that $\Phi_e(x, y)$ at the central region of the x-ray pulse (especially within the FWHM size of the x-ray pulse, black solid line) is sufficiently high for AuNPs to grow via diffusion-limited mechanism. By contrast, $\Phi_e(x, y)$ at the boundary (especially in between the black solid line and the $1/e^2$-size-boundary of the x-ray pulse (gray solid line)) is quite low, allowing the growth of AuNPs through a reaction-limited growth mechanism.

Method S3. Computational methods and details

S3.1. Density functional theory and solvation model

The hydration potential of electron and reduction potentials (RPs) of metal complexes were theoretically estimated by using density functional theory (DFT) calculations [3]. The DFT calculations were performed using Jaguar (9.1 suite) [4]. Geometries of the solvated structure of related species in solvent (water) were optimized using the Poisson-Boltzmann Finite element (PBF) method (ε = 78.4, r = 2.18) [5,6]. The Becke's 3 parameter exchange functional [7,8] with Grimme's D3 dispersion correction [9] (B3LYP-D3) was employed for these optimizations. The transition metals were modeled with the LACVP basis set [10-12], which includes the effective core potentials. The relativistic effect was not applied for first-row transition metals. The other atoms were modeled with the 6-31G(d,p) basis set [13]. The Cartesian coordinates of the related species' optimized geometries are described in Table S2. Frequency calculations were conducted at the same level of theory as the geometry optimizations. The vibrational frequencies of the optimized geometries are listed in Table S3. Zero-point vibrational energies and entropy corrections were taken from the frequency calculations. The single point calculations with the cc-pVTZ(-f) basis set [14], which includes Dunning's correlation-consistent triple-ζ quality and a double set of polarization functions, were performed to obtain more accurate energies of the optimized structures. Solvation

energies were considered by the self-consistent reaction field (SCRF) [6,15,16] with the dielectric constant (ε) of water to get the solvation correction energies. The resulting energy components (electronic energy, zero-point vibrational energy, entropy, and solvation free energy) for the optimized geometries are listed in Table S4.

S3.2. Computation of the hydration potential of an electron and reduction potentials of metal complexes

The hydration potential (HP) of an electron, $E^{\circ}(e^{-}_{gas}/e^{-}_{aq})$, is defined as the potential corresponding to the free energy change of the hydrated electron (the electron solvated in water) relative to the standard hydrogen electrode (E°_{SHE} = –4.281 V) [17] according to the following equation.

$$E^{\circ}(e^{-}_{gas}/e^{-}_{aq}) = E^{\circ}_{SHE} \quad (\Delta G_{e-gas/e-aq}/F)$$

$$E^{\circ}(e^{-}_{gas}/e^{-}_{aq}) = E^{\circ}_{SHE} - (\Delta G_{e^{-}_{gas}/e^{-}_{aq}}/F) \quad \text{(S5)}$$

변경된 필드 코드

where $\Delta G_{e^{-}_{gas}/e^{-}_{aq}}$ $\Delta G_{e-gas/e-aq}$ refers to the free energy change of the hydrated electron, and F is Faraday constant (23.0605 kcal/(mol·V)).

변경된 필드 코드

The RPs (E°([Ox]/[Red])) for various metal complexes relative to SHE were calculated using the following equation.

$$E^{\circ}([Ox]/[Red])_{exp\ or\ DFT} = E^{\circ}_{SHE} \quad (\Delta G_{[Ox]/[Red]}/nF)$$

$$E^{\circ}([Ox]/[Red])_{exp\ or\ DFT} = E^{\circ}_{SHE} - (\Delta G_{[Ox]/[Red]}/nF) \quad \text{(S6)}$$

변경된 필드 코드

where F is Faraday constant (23.0605 kcal/(mol·V)), [Ox]/[Red] refers to the redox couple, *n* refers to the number of electrons involved in the reaction, exp or DFT refers to “calculated by experimental values (exp) or density functional theory (DFT)”, and $\Delta G_{[Ox]/[Red]}$ is the Gibbs

free energy change calculated according to the following equation, Equation (S7), for the processes described in the following section.

$$\Delta G_{[Ox]/[Red]} = \Sigma G(Sol) \text{ for products} - \Sigma G(Sol) \text{ for reactants} \tag{S7}$$

where G(Sol) is the solvation-corrected Gibbs free energy of each optimized intermediate, which was computed according to the following equations:

$$G(Sol) = G(Gas) + G(Solv) \tag{S8}$$

~~$G(Gas) = H(Gas) - TnS(Gas)$~~ $G(Gas) = H(Gas) - T \cdot S(Gas)$

변경된 필드 코드

(S9)

$$H(Gas) = E(SCF) + ZPE \tag{S10}$$

where G(Gas) is the free energy of the optimized structure using the PBF method; G(Solv) is the solvation free energy; H(Gas) is the enthalpy in the optimized structure using PBF method; T is the temperature (298.15 K); S(Gas) is the entropy in the optimized structure using the PBF method. The solvent entropies are implicitly included to G(Solv) in the continuum model in Equation (S8); E(SCF) is the electronic self-consistent field energy computed from the SCF convergence in the optimized structure using the PBF method; and ZPE is the vibrational zero-point energy. Note that the entropy we refer to is specifically the vibrational, rotational, and translational entropies of the solute(s), except that the single-atom has only the translational entropy, which is calculated from Sackur-Tetrode equation [18].

S3.3. Processes considered for the computation of RPs of metal complexes

The general reduction process of metal complexes ($E^{\circ}([ML_x]^{m}/[ML_x]^{(m-n)})$), the reduction process for the metal nanocluster generation ($E^{\circ}([ML_x]^{m}/M_{bulk})$) and the reduction process for the neutral free metal atom generation ($E^{\circ}([ML_x]^{m}/M_{atom})$) are expressed as in Equation (S11), Equation (S12), and Equation (S13), respectively.

$$[ML_x]^m(aq) + ne^-_{gas} \rightarrow [ML_x]^{(m-n)}(aq) \quad (S11)$$

$$[M^X L_x]^m(aq) + Xe^-_{gas} \rightarrow M^0(s) + xL(aq) \quad (S12)$$

$$[M^X L_x]^m(aq) + Xe^-_{gas} \rightarrow M^0 + xL(aq)$$

$$[M^X L_x]^m(aq) + Xe^-_{gas} \rightarrow M^0(aq) + xL(aq)$$

(S13)

변경된 필드 코드

where $[ML_x]^{(m-n)}$ is the nth reduced state of $[ML_x]^m$, n refers to the number of electrons involved in the reaction, m refers to the total charge of $[ML_x]$, x refers to the number of ligands (L) that the metal complex has, X is the oxidation state of the metal (M), and M^0 refers to the free metal atom.

While $E^\circ([ML_x]^m/M_{atom})$ can be calculated using Equation (S6) with DFT values for Equation (S13), it can be also estimated using related experimental values as explained in the following. First, the free energy changes of Equation (S12) and Equation (S13) can be written as Equation (S14) and Equation (S15), respectively.

$$\Delta G_{(Equation\ (S12))} = \Delta G_{red} + \Delta G_{dis} + \Delta G_{subl} - \Delta G_{sol}$$

변경된 필드 코드

$$\Delta G_{(Equation\ (S12))} = \Delta G_{red} + \Delta G_{dis} + \Delta G_{subl} - \Delta G_{sol} \quad (S14)$$

$$\Delta G_{(Equation\ (S13))} = \Delta G_{red} + \Delta G_{dis} \quad (S15)$$

where ΔG_{red} refers to the free energy change of the reduction of the metal complex, ΔG_{dis} refers to the free energy change of the dissociation of the metal complex, ΔG_{subl} refers to the free energy change of the sublimation of the metal (M), and ΔG_{sol} refers to the free energy change of the solvation of the metal (M).

From Equation (S14) and Equation (S15), $E^\circ([M^X L_x]^m/M_{atom})$ can be written as follows.

$$E^\circ\left([M^X L_x]^m / M_{atom}\right) = E^\circ\left([M^X L_x]^m / M_{bulk}\right) - \left[(\Delta G_{subl} - \Delta G_{sol})/X\right]$$

변경된 필드 코드

$$E^\circ\left([M^X L_x]^m / M_{atom}\right) = E^\circ\left([M^X L_x]^m / M_{bulk}\right) - \left[(\Delta G_{subl} + \Delta G_{sol})/X\right] \quad (S16)$$

where X is the oxidation state of the metal (M), ΔG_{subl} refers to the free energy of sublimation of metal M, and ΔG_{sol} refers to the free energy of solvation. Equation (S16) allows for the estimation of $E^\circ([M^X L_x]^m/M_{atom})$ using experimental values of $E^\circ([M^X L_x]^m/M_{bulk})$, ΔG_{subl}, and ΔG_{sol}.

S3.4. Detailed explanation for rationalizing the experimental results

This section discusses a detailed explanation for Table S1, which compares $E^\circ(e^-_{gas}/e^-_{aq})$ and RPs of metal complexes. $E^\circ(e^-_{gas}/e^-_{aq})$ has been investigated by various studies. Based on existing equilibrium data and absolute proton hydration energies, $E^\circ(e^-_{gas}/e^-_{aq})$ was calculated as a function of temperature (–2.78 V vs SHE) [19]. $E^\circ(e^-_{gas}/e^-_{aq})$ was also obtained from the free energies of the following processes:

$$e^-_{gas} + H^+_{gas} \rightarrow e^-_{aq} + H^+_{aq} \quad \text{(S17)}$$

$$H^+_{gas} \rightarrow H^+_{aq} \quad \text{(S18)}$$

$E^\circ(e^-_{gas}/e^-_{aq})$ for 1 M in the gas phase (–2.71 V vs SHE) [20] can be derived from the free energies of Equation (S17) [21,22] and Equation (S18) [23]. $E^\circ(e^-_{gas}/e^-_{aq})$ was also roughly estimated with simple water cluster models ($[H_2O]_n$, n = 4, 6) described by DFT (–2.64 V and –2.59 V for n = 4, 6) [20]. Our own calculation using the same approach reproduced the reported values well (–2.97 V, –2.96 V for n = 4, 6). In summary, $E^\circ(e^-_{gas}/e^-_{aq})$ is estimated to lie between –2.7 ~ –2.9 V vs SHE.

To describe the correlation between the reductive tendency of metal complexes and the generation of metal nanoparticles, we calculated the reduction potentials (V vs SHE) and free energy changes that correspond to each metal complexes ($[AuCl_4]^-$, $[Au(tpy)Cl]^+$, $[Au(CN)_2]^-$, $[Pt_2(P_2O_5H_2)_4]^{4-}$, and $[Co(tpy)_2]^{2+}$) relative to SHE.

$E^{\circ}([M^{X}L_{x}]^{m}/M_{atom})$ can be estimated (as $E^{\circ}([M^{X}L_{x}]^{m}/M_{atom})_{exp}$) using the experimental values of $E^{\circ}([M^{X}L_{x}]^{m}/M_{bulk})$, ΔG_{subl}, and ΔG_{sol} according to Equation (S16). $E^{\circ}([M^{X}L_{x}]^{m}/M_{atom})$ is more negative than the reduction potential of the metal nanocluster generation ($E^{\circ}([M^{X}L_{x}]^{m}/M_{bulk})$) due to ΔG_{subl}. Therefore, a metal nanocluster cannot be generated without any metallic seeds if the potential of the electron hydration is higher than $E^{\circ}([M^{X}L_{x}]^{m}/M_{atom})$ [24,25]. $E^{\circ}([M^{X}L_{x}]^{m}/M_{atom})$ can be also calculated with DFT ($E^{\circ}([M^{X}L_{x}]^{m}/M_{atom})_{DFT}$). Both values ($E^{\circ}([M^{X}L_{x}]^{m}/M_{atom})_{exp}$, $E^{\circ}([M^{X}L_{x}]^{m}/M_{atom})_{DFT}$) for the reduction potential of the generation of a neutral atom are slightly different, but have a similar tendency as explained in the following.

For the gold complexes ($[AuCl_4]^{-}$, $[Au(tpy)Cl]^{+}$, and $[Au(CN)_2]^{-}$), ΔG_{subl} of gold has a value between 3.15 [26] ~ 3.67 [27] eV, and ΔG_{sol} of neutral gold atoms is regarded as –0.10 ~ 0.00 eV [26]. Some gold complexes ($[AuCl_4]^{-}$ and $[Au(tpy)Cl]^{+}$) that are observed to be rapidly reduced to metal clusters during TRXL experiments have higher reduction potentials than the potential of the electron hydration (–2.7 ~ –2.9 V). From experimental values (ΔG_{subl} = 3.67 eV, ΔG_{sol} ~ 0 eV, $E^{\circ}([Au^{III}Cl_4]^{-}/Au_{bulk})_{exp}$ = 1.00 V [28], $E^{\circ}([Au^{III}Cl_4]^{-}/Au_{bulk})_{exp}$ = 0.86 V) [29] and Equation (S16), $E^{\circ}([Au^{III}Cl_4]^{-}/Au_{atom})_{exp}$ and $E^{\circ}([Au^{III}(tpy)Cl]^{2+}/Au_{atom})_{exp}$ are calculated to –0.22 V vs SHE and –0.36 V vs SHE, respectively. From DFT, $E^{\circ}([Au^{III}Cl_4]^{-}/Au_{atom})_{DFT}$ and $E^{\circ}([Au^{III}(tpy)Cl]^{2+}/Au_{atom})_{DFT}$ are calculated to 0.44 V vs SHE and 0.07 V vs SHE, respectively. These values, which are higher than $E^{\circ}(e^{-}_{gas}/e^{-}_{aq})$, indicate that the gold complexes such as $[AuCl_4]^{-}$ and $[Au(tpy)Cl]^{+}$ can be reduced to metal nanocluster without any metallic seeds early in the radiolysis.

By contrast, metal complexes that hardly form the metal clusters during TRXL experiments such as ($[Au(CN)_2]^{-}$, $[Pt_2(P_2O_5H_2)_4]^{4-}$, and $[Co(tpy)_2]^{2+}$) have lower potential values. As the first example, $[Au(CN)_2]^{-}$, which does not generate gold nanoclusters within a few hours of a TRXL experiment, has a lower potential than $E^{\circ}(e^{-}_{gas}/e^{-}_{aq})$ (–2.7 ~ –2.9 V). From

서식 있음: 글꼴: 기울임꼴 없음

experimental values (ΔG_{subl} = 3.67 eV, ΔG_{sol} ~ 0 eV, E°([$Au^{I}(CN)_2]^{-}$/$Au_{bulk})_{exp}$ = –0.6 V[S24]) and Equation (S16), E°([$Au^{I}(CN)_2]^{-}$/$Au_{atom})_{exp}$ are calculated to –4.27 V. From DFT, E°([$Au^{I}(CN)_2]^{-}$/$Au_{atom})_{DFT}$ are calculated to –3.31 V. These values, which are lower than E°(e^{-}_{gas}/e^{-}_{aq}), indicate that [$Au^{I}(CN)_2]^{-}$ is not likely to be reduced to metal nanocluster in a few hours without any metallic seeds. As the second example, $[Pt_2(P_2O_5H_2)_4]^{4-}$ and $[Co(tpy)_2]^{2+}$ have higher potential than the electron hydration when they are not dissociated (E°($[Pt^{II}_2(P_2O_5H_2)_4]^{4-}$/$[Pt^{II}_2(P_2O_5H_2)_4]^{8-}$) = –2.16 V, E°($[Co^{II}(tpy)_2]^{2+}$/$[Co(tpy)_2]$) = –1.19 V). After reduction, however, $[Pt_2(P_2O_5H_2)_4]^{8-}$ and $[Co(tpy)_2]$ have free energy change of dissociation (ΔG_{dis}) of 257.23 kcal/mol (ΔG_{dis}($[Pt_2(P_2O_5H_2)_4]^{8-}$)) and 89.46 kcal/mol (ΔG_{dis}($[Co(tpy)_2]$)), respectively. Such high ΔG_{dis} makes E°([Ox]/[Red]) in Equation (S6) a largely negative value lower than E°(e^{-}_{gas}/e^{-}_{aq}) and thus would not allow their dissociation at room temperature, even after reduction. Consequently, metal nanoclusters cannot be generated from $[Pt_2(P_2O_5H_2)_4]^{4-}$ and $[Co(tpy)_2]^{2+}$.

서식 있음: 글꼴: 기울임꼴 없음

Supporting Tables

Table S1. Hydration potential (HP) of an electron, standard reduction potential (RP) of the molecule studied in this work, and the molecules containing metal studied in previous TRXL work, and free energy changes (ΔG) of related reactions. The dielectric constant of ε = 78.4 (water) was used when necessary.

#	Species	Reaction	HP or RP (V vs SHE)	ΔG (kcal/mol)
1	electron	$e^-_{gas} \rightarrow e^-_{aq}$	–2.78*[19]<br>–2.71*[20-23]	
		$e^-_{gas} + {}^{aq}[H_2O]_4 \rightarrow {}^{aq}[H_2O]_4^-$	–2.64***[20]<br>–2.97**	
		$e^-_{gas} + {}^{aq}[H_2O]_6 \rightarrow {}^{aq}[H_2O]_6^-$	–2.59***[20]<br>–2.96**	
2	$[Au^{III}Cl_4]^-$	$[Au^{III}Cl_4]^- + 3\ e^-_{gas} \rightarrow Au^0$ (atom) + $4Cl^-$	0.44**	
3	$[Au^{III}(tpy)Cl]^{2+}$	$[Au^{III}(tpy)Cl]^{2+} + 3\ e^-_{gas} \rightarrow [Au(tpy)Cl]^-$	0.11**	
		$[Au^0(tpy)Cl]^- \rightarrow Au^0$ (atom) + tpy + Cl^-		3.35**
		$[Au^0(tpy)Cl]^{2+} + 3\ e^-_{gas} \rightarrow Au^0$ (atom) + tpy + Cl^-	0.07**	
4	$[Au^I(CN)_2]^-$	$[Au^I(CN)_2]^- + e^-_{gas} \rightarrow [Au^0(CN)_2]^{2-}$	–2.58**	

		$[Au^0(CN)_2]^{2-} \rightarrow Au^0$ (atom) + 2 $(CN)^-$		16.69**
		$[Au^I(CN)_2]^- + e^-_{gas} \rightarrow Au^0$ (atom) + 2 $(CN)^-$	–3.31**	
5	$[Pt^{II}_2(P_2O_5H_2)_4]^{4-}$	$[Pt^{II}_2(P_2O_5H_2)_4]^{4-}$ + 4 $e^-_{gas} \rightarrow [Pt^0_2(P_2O_5H_2)_4]^{8-}$	–2.16**	
		$[Pt^{II}_2(P_2O_5H_2)_4]^{8-} \rightarrow$ 2 Pt^0 (atom) + 4 $(P_2O_5H_2)^{2-}$		257.23**
		$[Pt^{II}_2(P_2O_5H_2)_4]^{4-}$ + 4 $e^-_{gas} \rightarrow$ 2 Pt^0 (atom) + 4 $(P_2O_5H_2)^{2-}$	–4.95**	
6	$[Co^{II}(tpy)_2]^{2+}$	$[Co^{II}(tpy)_2]^{2+}$ + 2 $e^-_{gas} \rightarrow [Co(tpy)_2]$	–1.19**	
		$[Co^0(tpy)_2] \rightarrow Co^0$ (atom) + 2(tpy)		89.46**
		$[Co^{II}(tpy)_2]^{2+}$ + 2 $e^-_{gas} \rightarrow Co^0$ (atom) + 2(tpy)	–3.13**	

*Experimental values.

Calculated values from the same functional and basis set (B3LYP-D3/cc-pVTZ(-f)//PBF-B3LYP-D3/LACVP)

***Calculated by the G4 method with IEF-PCM (ε = 78.4) [20].

Table ~~S3~~S2. Cartesian coordinates of the optimized geometries.

===

$^{aq}[H2O]_4$

===

O	−1.852294445	0.702098608	0.105657049
H	−1.854121804	1.519870639	−0.419122189
O	−0.576326072	−1.241030812	−1.408554077
O	1.860900402	−0.406339556	−0.357224584
O	0.447142988	0.934587657	1.636790991
H	1.028143525	0.468162179	0.986566424
H	−1.092057824	0.827335298	0.725984156
H	1.046377659	−0.71737653	−0.822842598
H	2.241224289	−1.222483277	0.007894037
H	−0.727287591	−2.05617094	−0.901743114
H	−1.119081259	−0.563634992	−0.93186301
H	0.711442351	1.866568089	1.563789248

===

$^{aq}[H2O]_4^-$

===

O	−0.944653451	0.384108752	2.170994043
H	−1.840880513	0.654699683	1.906993985
O	−1.562235236	−1.426666856	−1.341658473
O	0.386327267	2.121998072	−1.208958149
O	2.266937494	−1.154979944	0.282805979
H	1.371682525	−0.74638778	0.117408775

H	−0.509210348	0.211576149	1.287005305
H	0.290290087	1.254019022	−0.723357797
H	−0.519127488	2.476143837	−1.173292398
H	−1.55569911	−2.228960037	−0.791866064
H	−0.92010051	−0.836149991	−0.856040776
H	2.732658386	−0.444250464	0.756126523

===

[aq][H2O]$_6$

===

H	−0.180244282	0.22419472	2.418284416
H	2.506551743	−0.105846353	−0.234925434
H	−0.194529936	2.516952276	0.141183943
H	0.163385347	−0.242701009	−2.418646336
H	−2.471465826	0.123821564	0.260842741
H	0.193514466	−2.511047602	−0.1639238
O	−0.555158615	−2.340545416	0.430965185
O	−2.30729723	−0.443156004	−0.510912061
O	0.411919981	−0.528555334	2.256408691
O	−0.419448048	0.518072307	−2.260299921
O	0.556356966	2.335840464	−0.44812873
O	2.312684059	0.458252817	0.531681955
H	1.130962491	−0.171331331	1.678300261
H	−0.195330635	−1.748494625	1.13711524
H	−1.737172246	−1.17525959	−0.16883716
H	−1.140600443	0.171644673	−1.678222179
H	0.194932029	1.732620239	−1.144261003

H	1.744963527	1.186893344	0.177610502

==

aq$[H2O]_6^-$

==

O	1.611056209	0.246199057	2.008482933
O	−2.522862673	0.741017342	0.930523634
O	0.927244544	2.320927143	−1.0222224
O	2.676747084	−0.013940183	−0.771415055
O	−2.457681417	−2.166127205	0.397025257
H	0.300110519	1.721817851	−0.510810077
H	0.754391432	0.435427755	1.559661984
H	−1.555497885	0.788498044	0.639573455
H	−1.647551417	−2.100523472	−0.141499579
H	2.294684649	0.878430188	−0.905767918
H	−2.998052835	0.994559467	0.118244931
H	0.622881353	2.220093966	−1.94266808
H	1.900426507	−0.583507836	−0.946942568
H	−2.593978882	−1.23529923	0.667067707
H	2.201275587	0.167678267	1.233763814
H	−0.299556732	−1.062781811	−1.982715964
O	0.019731142	−1.269360304	−1.083937526
H	−0.208870053	−0.437451333	−0.558606565

==

CN^-

==

C	0.899958491	−0.033039216	−0.961959183
N	0.899958491	−0.033039216	0.221266583

==

Terpyridine(tpy)

==

C	0.533415139	0.403907657	−5.163493633
C	1.260860085	5.112699986	−0.783003807
C	0.712073028	3.925187111	−0.290471077
N	0.590107024	2.801879883	−1.012164593
C	1.019038677	2.82611227	−2.29227829
C	1.591543317	3.971411467	−2.869409561
C	1.71259737	5.130512238	−2.103112698
H	0.350383639	3.876551151	0.735254049
H	1.958575487	3.951031685	−3.890999794
H	2.158149004	6.025729179	−2.529051304
H	1.332392335	5.989588737	−0.145207331
C	0.876029193	1.564662933	−3.080886841
N	0.979820788	0.408631444	−2.400049686
C	0.877122819	−0.748212039	−3.078788042
C	0.652152598	−0.795391977	−4.464426994
C	0.648434818	1.605157256	−4.467170238
H	0.545846105	−1.746930599	−4.97664547
H	0.34069258	0.402499616	−6.233119011
H	0.536280453	2.552757025	−4.984930515
C	1.015444517	−2.008354664	−2.288526773
N	0.547996938	−1.994758248	−1.022097945

C	0.665333688	−3.120168924	−0.302405596
C	1.246767998	−4.296807289	−0.784278274
C	1.737860322	−4.302166462	−2.090626478
C	1.620563865	−3.141407728	−2.854969501
H	0.272556067	−3.082476616	0.712147355
H	1.312635422	−5.17548275	−0.148327053
H	2.210002422	−5.188154221	−2.507013321
H	2.014763355	−3.107207298	−3.865978718

==

$[P_2O_5H_2]^{2-}$

==

H	0.085240826	−0.89269489	−2.689137936
O	−0.806760967	−0.985705376	−3.074457407
P	−1.986564517	−1.012931228	−1.828909159
O	−1.441751361	−2.035875082	−0.784386516
O	−1.689711571	0.521365762	−1.160126925
P	−0.46843186	0.960154891	0.014458687
O	−1.10945034	0.679903805	1.389225245
O	0.604543984	−0.332781821	−0.313462913
H	0.042946566	−1.153561234	−0.350527316

==

$[AuCl_4]^{-}$

==

Au	−0.389142931	−0.005980418	−1.481775522
Cl	−0.391093969	2.384328127	−1.490731597

Cl	−0.391196549	−2.395922661	−1.475910902
Cl	−2.779374123	−0.003800763	−1.490238309
Cl	2.000772238	−0.003825143	−1.475280643

===

$[Au(tpy)Cl]^{2+}$

===

C	1.016032815	0.406967759	−5.400130749
C	1.015726447	4.754375458	−0.461694956
C	1.015967727	3.404141665	−0.109062657
N	1.016166925	2.45090127	−1.053937078
C	1.01607573	2.763139009	−2.394826412
C	1.015892267	4.097429276	−2.788080692
C	1.015690565	5.101500034	−1.811103463
H	1.015976787	3.077269793	0.924806654
H	1.015841722	4.357458591	−3.842533588
H	1.015475035	6.146617413	−2.110327482
H	1.015547752	5.505298138	0.323077053
C	1.016111374	1.612962127	−3.320827723
N	1.016216516	0.408616722	−2.708669424
C	1.016277313	−0.796554744	−3.318336964
C	1.016263127	−0.814839602	−4.715672016
C	1.015952468	1.629965544	−4.717186928
H	1.016379237	−1.753773212	−5.260965824
H	1.015876174	0.405872017	−6.487038612
H	1.01569593	2.569103718	−5.262477398
C	1.016207337	−1.946574569	−2.391142607

N	1.016252995	−1.635273695	−1.05011487
C	1.015976906	−2.588657141	−0.107375965
C	1.015573025	−3.93888092	−0.460092008
C	1.015584469	−4.286024094	−1.809896946
C	1.015943289	−3.280661106	−2.786169052
H	1.016061783	−2.262451172	0.927264512
H	1.015172243	−4.689342976	0.325225323
H	1.01527679	−5.331032276	−2.1095438
H	1.015949249	−3.539550066	−3.840690613
Au	1.016410232	0.409630597	−0.720787764
Cl	1.016563773	0.410436273	1.636713982

===

$[Au(CN)_2]^-$

===

Au	1.127040029	1.18434E−4	−1.454093575
C	2.075099468	−0.218034178	0.302748322
C	0.179727197	0.217533171	−3.211372852
N	−0.371860534	0.344182402	−4.235056877
N	2.627234697	−0.34541896	1.326045156

===

$[Au(CN)_2]^{2-}$

===

Au	1.126873255	1.69319E−4	−1.45397675
C	2.068963766	−0.216567799	0.290781647
C	0.186511591	0.215852037	−3.199714899

N	−0.370620042	0.343892902	−4.235007286
N	2.627782822	−0.345661134	1.324616671

==

$[Pt_2(P_2O_5H_2)_4]^{4-}$

==

Pt	−1.249643326	−0.448024929	0.385401607
P	−2.170010328	1.688783646	0.783799648
P	−2.077373743	−0.388947845	−1.824907184
P	−0.43077141	−0.517271638	2.599287748
P	−0.350664258	−2.595820665	−0.007697124
O	−1.298157334	−3.492178202	−0.9668203
O	0.020747982	−3.471599579	1.194803834
O	0.9869771	−2.475184917	−0.997173905
P	2.21247983	−1.340916991	−0.935827315
O	2.779457331	−1.500404358	−2.443288803
O	3.299274445	−1.865238786	0.007972605
Pt	1.295326948	0.797095001	−0.537053466
P	0.475434512	0.864025056	−2.751092672
P	2.12280798	0.735325933	1.673495531
P	0.396148145	2.946098566	−0.146141022
O	1.23095417	−0.35233587	2.576116085
O	−0.526126623	−1.997517467	3.248919725
O	1.038612962	−0.118198678	−3.782559395
O	−0.995520234	0.463630557	3.633223534
O	−1.18830514	0.700205624	−2.72529459
O	2.216572046	2.044931173	2.463464022

O	1.33452487	3.842833042	0.822016478
O	0.568679452	2.344284296	−3.400976181
O	3.568377495	0.013378681	1.773876905
O	−2.17389226	−1.69866097	−2.614191055
O	−3.525306463	0.33121714	−1.920940161
O	−0.950950384	2.827979565	0.833862662
O	0.030445248	3.823799133	−1.348878503
O	−3.267156839	2.207550049	−0.152521014
O	−2.730113983	1.850489259	2.295476913
H	2.158370733	−1.003772855	−3.071962118
H	−0.313057244	−2.672610998	2.519712448
H	0.361117184	3.021995068	−2.672331095
H	3.579034805	−0.76160717	1.119532824
H	−3.534646988	1.107427359	−1.266787291
H	−1.691671014	−2.885427713	−1.679208994
H	−2.106915712	1.350143313	2.92057395
H	1.733708739	3.232581615	1.527860045

==

$[Pt_2(P_2O_5H_2)_4]^{8-}$

==

Pt	−1.457527995	−0.596928656	0.467392117
P	−1.769000053	1.937146664	0.567537487
P	−2.498731375	−0.641803682	−1.605142713
P	−0.883531094	−0.77616483	2.705049276
P	0.155723244	−2.486078739	−0.107971638
O	−0.590351164	−3.382554531	−1.359445453

O	0.352806121	−3.553827286	1.028388262
O	1.724926353	−2.477086067	−0.869915128
P	2.655521631	−1.097608685	−1.07899785
O	2.993891239	−1.286272168	−2.702321291
O	3.99622488	−1.442434549	−0.371597648
Pt	1.530718327	0.873637736	−0.60264653
P	0.084120877	0.528394461	−2.667263031
P	1.702859521	0.589439511	1.91674614
P	0.883855045	3.07506156	−0.303071022
O	0.644727588	−0.186493814	3.06265974
O	−0.665885389	−2.351844549	3.207127094
O	0.795387924	−0.224107072	−3.847512484
O	−1.775933385	−0.149687141	3.813280582
O	−1.560202241	−0.017198838	−2.844424963
O	2.116624594	1.887816429	2.699078321
O	1.759898901	3.85130167	0.888071001
O	−0.212737098	2.079759598	−3.314576864
O	3.015468597	−0.46953401	2.19704628
O	−3.034191847	−1.975118518	−2.196285725
O	−3.796144485	0.39840427	−1.718805313
O	−0.639564633	3.252304316	0.365124047
O	0.89736855	4.077494621	−1.489762306
O	−3.038171768	2.477737427	−0.181222513
O	−2.098806858	2.307615519	2.20697999
H	2.208365202	−0.88435334	−3.190545559
H	−0.293841869	−2.850960493	2.414118052
H	0.232363522	2.73083663	−2.725437403

H	3.363893032	−0.731684566	1.313480973
H	−3.569128513	1.197211742	−1.148689747
H	−1.436191201	−2.925800562	−1.572563529
H	−2.053046942	1.46057713	2.704674959
H	1.941500306	3.15135169	1.590064764

==

$[Co(tpy)_2]^{2+}$

==

C	1.222643495	1.751266837	−1.085292339
C	1.133079886	6.771680355	3.0862813
C	1.143611908	5.501709938	3.66528368
N	1.157846928	4.380016327	2.938374043
C	1.163809538	4.456633568	1.589339972
C	1.152630925	5.692698479	0.932623386
C	1.13737762	6.861771584	1.694419622
H	1.139299035	5.374398232	4.745251179
H	1.156264901	5.756534576	−0.150344864
H	1.129107833	7.83046627	1.201308608
H	1.122094512	7.658656597	3.713409185
C	1.186559677	3.148608685	0.875661552
N	1.202008486	2.044745207	1.662344575
C	1.228098392	0.799476206	1.125561595
C	1.240026474	0.625838757	−0.263223559
C	1.195507526	3.024564028	−0.518536091
H	1.265645266	−0.365850389	−0.702159882
H	1.232397556	1.636102796	−2.166364908

H	1.183259487	3.901494741	−1.156891823
C	1.246295571	−0.328828573	2.097381353
N	1.267627597	0.029302435	3.400045395
C	1.288916349	−0.915430784	4.344893456
C	1.287942886	−2.278576612	4.044691563
C	1.26237607	−2.658823967	2.702920437
C	1.241304994	−1.675267577	1.713323832
H	1.306233048	−0.564870477	5.374018669
H	1.305570245	−3.014576435	4.843410492
H	1.257979155	−3.709611654	2.424757719
H	1.218861699	−1.9632622	0.667531967
C	1.300428748	2.722302914	8.221113205
C	−3.121326447	2.06832695	3.434737682
C	−1.778009295	2.0912714	3.059558153
N	−0.788993597	2.22150445	3.954387903
C	−1.093748689	2.33625865	5.277653217
C	−2.415687799	2.320905447	5.724370956
C	−3.441890001	2.184761047	4.787432194
H	−1.479453444	2.004631996	2.019630909
H	−2.645068407	2.411712408	6.782066345
H	−4.478517056	2.16975522	5.113721848
H	−3.891719818	1.960339069	2.676849842
C	0.084476389	2.469658852	6.163249493
N	1.255759001	2.44295764	5.492206097
C	2.448439121	2.546741724	6.11728096
C	2.501737833	2.690092564	7.507396221
C	0.076747924	2.611631632	7.554673195

H	3.451985359	2.774696112	8.025738716
H	1.318148971	2.833540916	9.302459717
H	−0.855856717	2.636526108	8.109869957
C	3.597290277	2.489647865	5.18773222
N	3.249251366	2.341564894	3.87842083
C	4.21152544	2.27735877	2.949440002
C	5.566191196	2.356189728	3.27051878
C	5.930269241	2.507260323	4.60824585
C	4.933078766	2.575190067	5.58286953
H	3.883882523	2.159208298	1.921599388
H	6.311252594	2.299388885	2.482412338
H	6.977077961	2.571762085	4.893484592
H	5.195730209	2.692658186	6.630072117
Co	1.225789428	2.249401093	3.605635166

==

[Co(tpy)$_2$]

==

C	1.707928181	1.665124655	−1.251723766
C	0.863142252	6.704493999	2.847342968
C	0.839268029	5.429778099	3.40920043
N	0.986886024	4.31292963	2.684687614
C	1.171054602	4.403865337	1.347041249
C	1.206044793	5.651433945	0.706381083
C	1.050469041	6.810786724	1.464718461
H	0.699134409	5.279868126	4.477255344
H	1.356505156	5.71985817	−0.366494894

H	1.078125119	7.785572052	0.983646333
H	0.73971194	7.583652973	3.473985434
C	1.338947535	3.100527525	0.645029366
N	1.304768562	2.032148361	1.469417095
C	1.44928205	0.750943542	0.978069067
C	1.667215228	0.567442536	−0.411312073
C	1.534311533	2.973636389	−0.725934565
H	1.798192501	−0.431728929	−0.818010747
H	1.870697498	1.528419137	−2.317979336
H	1.559103608	3.837625265	−1.380746841
C	1.327169776	−0.295350999	1.962018847
N	1.100571036	0.133485079	3.259232521
C	0.92080605	−0.774542511	4.232047081
C	0.969513297	−2.145662546	4.023968697
C	1.219813943	−2.604150534	2.704186678
C	1.393337727	−1.688518286	1.685362935
H	0.742383361	−0.358163804	5.221989632
H	0.831790984	−2.836224318	4.850806236
H	1.271867752	−3.670048475	2.493126154
H	1.575666428	−2.034011126	0.671164155
C	1.446661234	2.443899155	8.350084305
C	−3.35230875	2.255322933	3.905361652
C	−2.051402807	2.298305273	3.40931797
N	−0.970843494	2.359372854	4.199939728
C	−1.129559636	2.376696348	5.546180725
C	−2.41067338	2.338089705	6.122968674
C	−3.528279066	2.277565241	5.295508385

H	−1.848769069	2.284748554	2.340703726
H	−2.534144163	2.349833965	7.201684475
H	−4.525559902	2.244580746	5.728004932
H	−4.199275017	2.206484318	3.226309776
C	0.129334435	2.418629408	6.329336166
N	1.26068449	2.403876543	5.569049835
C	2.500500202	2.415886164	6.172577858
C	2.600285053	2.428148508	7.576441765
C	0.182282344	2.442586184	7.716768742
H	3.573610067	2.42813158	8.05910778
H	1.513220906	2.457312107	9.434713364
H	−0.72334832	2.453892231	8.31467247
C	3.627142668	2.44002676	5.255950928
N	3.298242092	2.42652297	3.919642925
C	4.280806541	2.484032631	3.001801014
C	5.628006458	2.541954279	3.32659173
C	5.981870651	2.54413271	4.696231365
C	4.984982014	2.496166468	5.6543293
H	3.946151257	2.471288919	1.966808915
H	6.382467747	2.575022459	2.545865774
H	7.026311398	2.585322618	4.996880531
H	5.246888161	2.504729509	6.70862627
Co	1.181460619	2.257158995	3.533944607

Table S43. Vibrational frequencies (in cm^{-1}) of the optimized structures.

==

$^{aq}[H_2O]_4$

==

79.55	92.50	205.74	247.63	254.12	259.07
368.02	382.60	413.34	425.33	501.49	532.38
581.76	639.22	718.39	843.73	886.85	1097.07
1706.68	1721.40	1723.52	1734.41	3319.14	3429.82
3437.81	3482.22	3777.82	3779.22	3781.36	3783.12

==

$^{aq}[H_2O]_4^-$

==

60.60	67.47	98.86	101.55	104.92	135.05
164.05	200.53	224.77	232.48	342.17	370.69
379.70	402.91	523.03	552.32	600.10	630.45
1592.39	1624.53	1641.33	1645.86	3123.56	3154.76
3175.33	3267.29	3707.03	3708.31	3716.56	3721.32

==

$^{aq}[H_2O]_6$

==

17.20	19.93	61.79	62.74	67.85	68.29
211.35	241.83	245.23	265.51	270.56	272.82
350.86	353.77	363.48	370.23	392.12	435.19

496.37	501.26	522.93	530.84	556.27	561.73
780.94	792.07	799.00	866.50	882.06	990.05
1682.06	1703.35	1704.79	1738.01	1740.30	1755.95
3305.15	3377.02	3378.72	3443.43	3447.34	3483.25
3778.73	3779.90	3781.68	3782.44	3783.34	3785.32

==

$^{aq}[H_2O]_6^-$

==

41.84	69.24	77.58	102.57	108.14	116.59
125.76	144.84	149.47	162.17	189.20	202.91
281.11	294.36	320.95	345.04	349.47	393.37
481.25	521.01	541.58	543.57	576.14	602.82
668.32	699.25	730.75	760.79	813.19	827.96
1517.33	1596.06	1610.97	1725.86	1743.63	1754.46
2892.64	2954.61	3051.50	3486.37	3584.22	3610.72
3633.84	3653.15	3666.00	3677.34	3688.64	3704.20

==

CN^-

==

2142.09

==

Terpyridine(tpy)

==

55.68	61.11	69.51	111.65	115.77	151.63
236.91	259.68	281.85	339.03	385.77	410.91
424.38	429.09	459.99	534.67	549.19	622.01
628.49	653.37	665.07	672.84	734.84	762.48
765.33	765.60	788.03	817.16	846.74	851.82
912.65	913.19	931.35	980.38	980.89	1000.08
1001.05	1001.41	1004.98	1010.10	1010.24	1058.45
1071.56	1097.00	1116.88	1126.83	1132.30	1152.16
1188.50	1190.73	1208.59	1288.65	1288.98	1292.35
1296.25	1309.66	1329.80	1348.22	1428.95	1464.01
1478.83	1479.77	1506.22	1515.03	1610.27	1616.47
1624.74	1629.18	1632.31	1633.88	3162.35	3163.54
3178.84	3179.88	3181.03	3193.39	3195.23	3198.78
3203.27	3205.64	3207.96			

==

$[P_2O_5H_2]^{2-}$

==

62.54	97.45	215.58	259.07	332.62	340.32
386.82	442.40	504.87	541.94	560.82	642.18
708.62	755.53	896.98	1003.04	1071.09	1093.68
1275.45	3270.61	3660.35			

==

$[AuCl_4]^{-}$

==

68.88	120.00	141.02	141.04	144.63	272.64
300.33	318.86	318.88			

===

$[Au(tpy)Cl]^{2+}$

===

49.24	80.32	95.84	118.07	133.14	143.83
146.14	208.06	229.61	237.48	279.07	284.78
286.20	334.56	361.61	365.24	421.37	430.66
454.39	458.73	474.38	519.92	556.03	633.68
652.57	666.08	676.59	692.49	730.13	739.45
754.83	756.35	804.47	808.74	839.14	850.15
926.02	930.47	949.85	1015.34	1016.47	1032.67
1042.72	1045.79	1050.91	1052.29	1052.66	1074.37
1081.08	1097.49	1123.77	1131.29	1152.71	1176.95
1213.59	1216.47	1231.45	1305.63	1313.89	1318.28
1336.16	1341.30	1365.22	1371.34	1436.51	1478.67
1493.64	1494.80	1523.77	1539.04	1609.14	1613.30
1616.27	1624.32	1634.18	1637.58	3205.31	3206.06
3207.82	3214.04	3216.05	3219.73	3221.14	3222.18
3223.99	3234.88	3239.77			

===

$[Au(CN)_2]^-$

===

80.17	80.24	294.07	294.31	411.43	415.03
415.35	426.76	2246.50	2258.57		

==

$[Au(CN)_2]^{2-}$

==

91.37	101.02	352.53	380.05	419.59	430.89
438.91	456.07	2141.56	2149.73		

==

$[Pt_2(P_2O_5H_2)_4]^{4-}$

==

48.33	62.77	77.01	77.53	81.89	85.85
92.22	110.63	125.82	139.11	149.22	165.21
166.60	172.76	174.12	186.79	218.54	219.83
220.15	223.48	224.77	234.10	237.08	237.97
251.28	252.18	254.63	268.63	270.65	284.16
285.50	289.24	291.85	293.27	306.70	312.05
319.24	324.64	325.80	326.49	336.41	337.10
339.33	340.96	392.31	402.49	403.01	434.14
445.69	449.37	452.83	459.81	495.14	501.19
503.62	504.45	512.79	515.77	535.41	546.86
710.34	711.16	712.19	732.13	811.62	812.33
818.35	874.20	875.25	876.07	876.63	885.84
886.34	888.60	890.34	900.29	903.09	905.18

907.10	912.90	915.05	916.53	920.33	924.82
1076.08	1091.79	1094.06	1095.64	1106.84	1111.43
1113.81	1128.85	1404.57	1407.55	1409.63	1410.56
1413.13	1416.42	1417.08	1420.89	2834.84	2837.56
2868.52	2880.40	2886.22	2901.53	2915.35	2946.95

==

$[Pt_2(P_2O_5H_2)_4]^{8-}$

==

114.50	116.15	118.06	135.40	148.08	150.97
152.76	153.43	208.85	212.72	217.98	218.64
219.75	224.38	227.30	229.69	235.24	235.77
236.67	237.57	251.76	252.98	253.11	253.75
271.75	272.41	273.30	281.28	317.67	318.59
319.70	320.72	375.56	377.05	380.45	387.97
397.47	399.93	401.58	403.34	454.23	456.03
456.70	458.77	473.13	473.41	486.89	501.49
622.44	631.07	634.41	663.24	673.91	677.32
678.59	682.41	688.54	692.60	699.23	702.30
755.58	758.36	760.42	774.89	779.37	780.46
784.62	795.39	830.62	832.26	841.44	845.26
964.90	966.04	973.14	974.26	1027.46	1029.18
1030.64	1036.71	1286.48	1293.61	1294.37	1300.12
1430.42	1435.20	1438.05	1439.15	3012.65	3019.43
3024.99	3032.11	3440.25	3452.94	3456.31	3474.75

==

$[Co(tpy)_2]^{2+}$

==

30.02	33.32	36.87	48.00	51.93	77.08
93.20	95.40	97.95	113.89	114.34	114.80
144.56	150.14	166.21	173.70	220.25	231.71
238.96	241.92	265.04	282.79	287.65	300.47
332.86	344.65	353.96	354.34	388.01	418.39
422.38	436.11	438.33	444.44	457.87	461.32
465.67	467.62	468.04	525.69	528.83	542.26
552.71	642.51	645.00	647.03	651.73	657.37
658.35	662.81	664.13	688.22	692.41	739.26
742.18	753.26	755.70	757.89	763.43	765.75
771.79	800.07	800.56	813.32	817.54	845.57
850.51	851.74	853.73	916.09	918.34	920.42
921.66	939.65	940.13	990.42	990.60	992.07
992.45	1023.03	1026.09	1028.88	1029.90	1035.02
1035.91	1036.26	1036.97	1037.36	1037.75	1052.40
1060.71	1063.86	1069.09	1076.71	1080.31	1097.31
1099.62	1126.76	1128.47	1128.75	1137.10	1146.99
1149.53	1169.53	1172.19	1204.11	1205.00	1207.76
1209.57	1226.15	1234.63	1301.35	1301.84	1305.44
1309.01	1312.02	1317.15	1323.38	1323.52	1331.89
1334.05	1345.31	1355.11	1357.82	1364.99	1436.34
1439.21	1478.67	1482.65	1488.84	1489.87	1494.96
1499.63	1513.86	1514.75	1529.03	1535.51	1610.11

1610.93	1620.55	1623.15	1626.36	1628.59	1636.90
1639.99	1641.61	1648.96	1649.40	1654.13	3183.22
3184.18	3196.92	3197.44	3198.04	3198.42	3199.23
3199.26	3206.42	3207.37	3211.16	3211.83	3212.21
3212.67	3216.38	3216.75	3217.30	3219.56	3221.70
3222.31	3226.62	3228.35			

===

[Co(tpy)$_2$]

===

29.49	30.83	35.50	43.26	45.72	77.98
81.83	85.59	94.45	100.96	111.97	117.52
136.77	142.10	143.21	147.27	193.67	197.50
213.95	224.15	233.09	244.16	249.70	250.18
258.49	303.75	318.55	354.07	356.47	394.15
398.43	420.48	422.01	427.79	431.77	436.28
443.80	458.68	459.96	523.80	529.30	535.32
536.70	618.56	621.55	627.70	627.84	639.31
641.03	657.40	658.40	665.90	669.39	698.82
699.27	724.80	731.11	732.46	734.75	743.65
749.91	760.70	765.63	773.12	782.76	804.02
806.05	841.72	843.96	846.80	851.08	865.70
878.48	894.57	896.06	930.07	939.49	940.32
949.24	964.28	968.87	973.08	974.18	974.50
980.99	989.03	994.54	995.47	997.00	1020.44
1025.00	1041.71	1048.76	1057.30	1060.17	1076.13

1077.79	1087.07	1091.73	1097.35	1104.94	1114.89
1117.07	1136.93	1142.82	1161.36	1163.11	1186.83
1188.32	1193.83	1195.90	1245.41	1261.44	1291.83
1295.03	1308.13	1311.80	1314.11	1316.20	1319.25
1320.73	1336.65	1344.81	1358.32	1370.29	1370.94
1386.48	1420.22	1442.53	1464.69	1465.67	1477.80
1482.22	1482.83	1485.70	1499.31	1510.04	1533.95
1540.84	1553.30	1564.63	1584.57	1602.04	1606.45
1610.41	1617.09	1620.15	1625.65	1628.18	3163.60
3167.92	3169.94	3172.55	3173.21	3175.05	3176.18
3178.60	3180.21	3182.06	3186.09	3189.42	3194.69
3195.17	3198.79	3198.93	3201.55	3202.14	3203.34
3204.55	3209.27	3214.18			

Table S24. Computed energy components for DFT-optimized geometries.

	E(SCF)/(eV)	ZPE/(kcal/mol)	S(gGas)/(cal/mol·K)	G(sSolv)/(kcal/mol)
	cc-pVTZ(-f) /LACV3P**	6-31G** /LACVP**	6-31G** /LACVP**	6-31G** /LACVP**
$^{aq}[H_2O]_4$	−8323.683	63.20	87.17	−20.34
$^{aq}[H_2O]_4^-$	−8322.193	56.14	105.12	−72.43
$^{aq}[H_2O]_6$	−12485.495	94.07	121.54	−29.87
$^{aq}[H_2O]_6^-$	−12484.912	89.77	122.38	−69.22
Cl^-	−12525.338	0.00	36.63	−81.96
CN^-	−2527.392	3.06	47.05	−80.35
Terpyridine(tpy)	−20210.150	142.08	115.27	−18.41
$[P_2O_5H_2]^{2-}$	−28853.164	25.91	88.10	−218.94
Au^0	−3685.613	0.00	41.74	0.00
Pt^0	−3239.749	0.00	41.71	0.00
Co^0	−3946.108	0.00	38.14	0.00
$[AuCl_4]^-$	−53786.180	2.61	94.71	−49.52
$[Au(tpy)Cl]^{2+}$	−36406.332	146.32	126.58	−164.46
$[Au(tpy)Cl]^-$	−36423.527	140.75	145.08	−60.92
$[Au(CN)_2]^-$	−8744.933	9.90	75.49	−55.39

$[Au(CN)_2]^{2-}$	−8740.650	9.95	75.20	−193.47
$[Pt_2(P_2O_5H_2)_4]^{4-}$	−121912.938	115.87	196.01	−541.58
$[Pt_2(P_2O_5H_2)_4]^{8-}$	−121852.766	110.252	220.44	−2112.13
$[Co(tpy)_2]^{2+}$	−44361.449	290.011	183.88	−129.29
$[Co(tpy)_2]$	−44372.293	283.285	191.16	−12.82

Supporting Figures

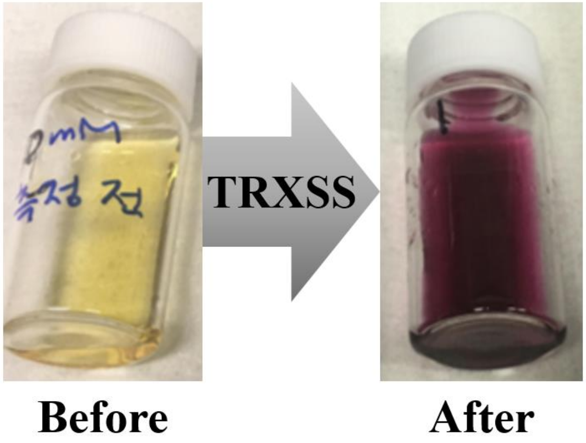

Figure ~~S2~~S1. Comparison of sample states before and after the TRXL experiment. The sample prepared before the TRXL experiment showed a transparent yellow color, and the sample after TRXL turned into a dark purple.

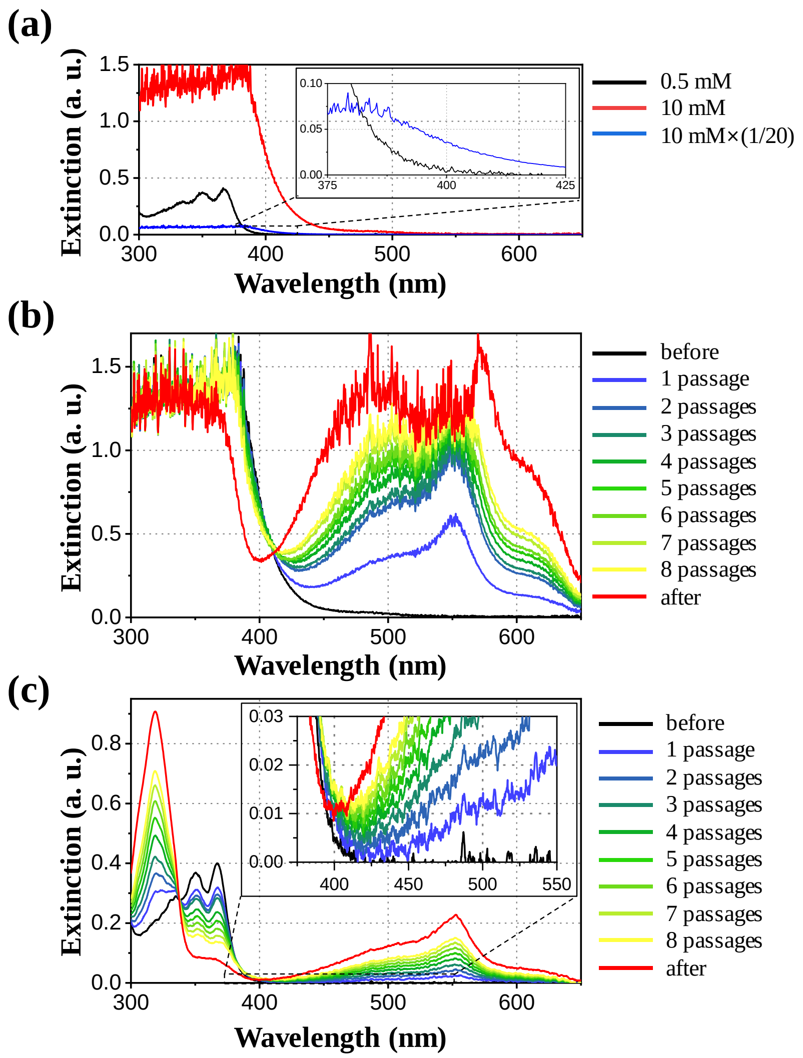

Figure ~~S3~~S2. Evolution of UV-visible spectra of the sample solution upon exposures to the laser and x-ray pulses. (a) The spectra of the sample before exposure to the laser and x-ray pulses. The spectra of the sample before (10 mM concentration, red) and after (20 times diluted to 0.5 mM concentration, black) a dilution are shown. The extinction value for the sample before the dilution is saturated and measured to be around 1.5 for the wavelengths shorter than 375 nm due to the measurement limit of the instrument. For the comparison of the spectra

before and after the dilution, the spectrum of the sample before the dilution which is scaled down by a factor of 1/20 is also shown (blue). The inset plot shows an enlarged view of the dashed box. Note that the shape of the spectrum changes as the concentration of the sample changes. (b-c) The spectra of the samples that passed through the x-ray and laser interaction region from one to eight times are shown. The spectra of the sample before (black) and after (red) the TRXL experiment are displayed together. The spectra were measured before (b) and after (c) diluting the samples. A more detailed procedure is described in the main text. The inset plot shows an enlarged view of the dashed box.

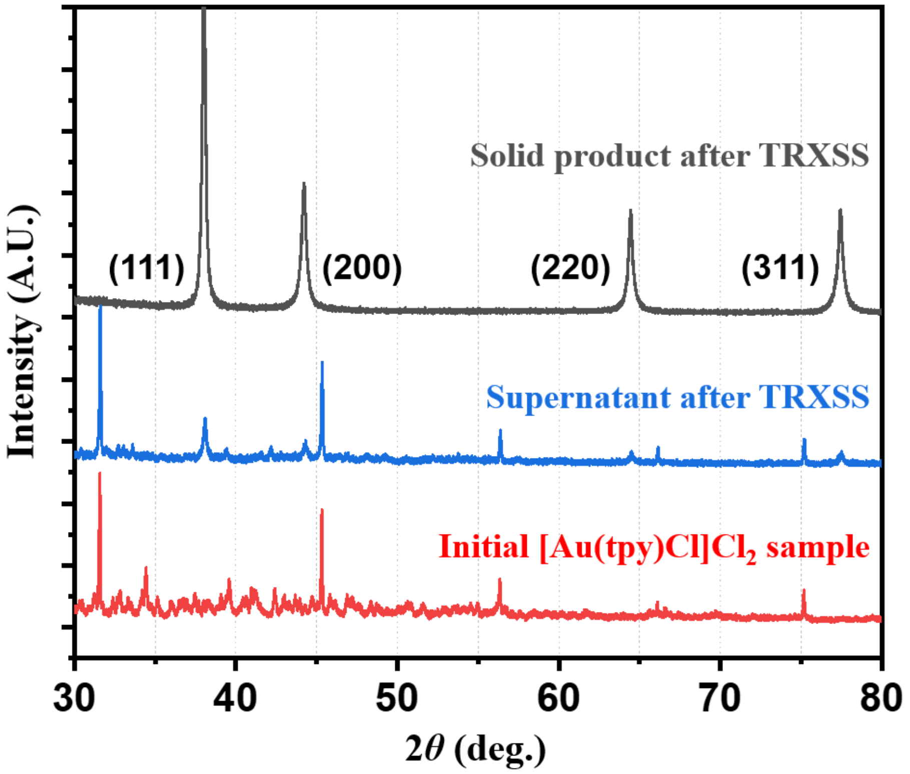

Figure ~~S4~~S3. PXRD patterns from the initial sample ([Au(tpy)Cl]Cl_2), the supernatant, and the solid product after the TRXL experiment. The solution sample after the TRXL experiment was centrifuged. The solid precipitate that is the solid product and the supernatant was taken and dried for PXRD.

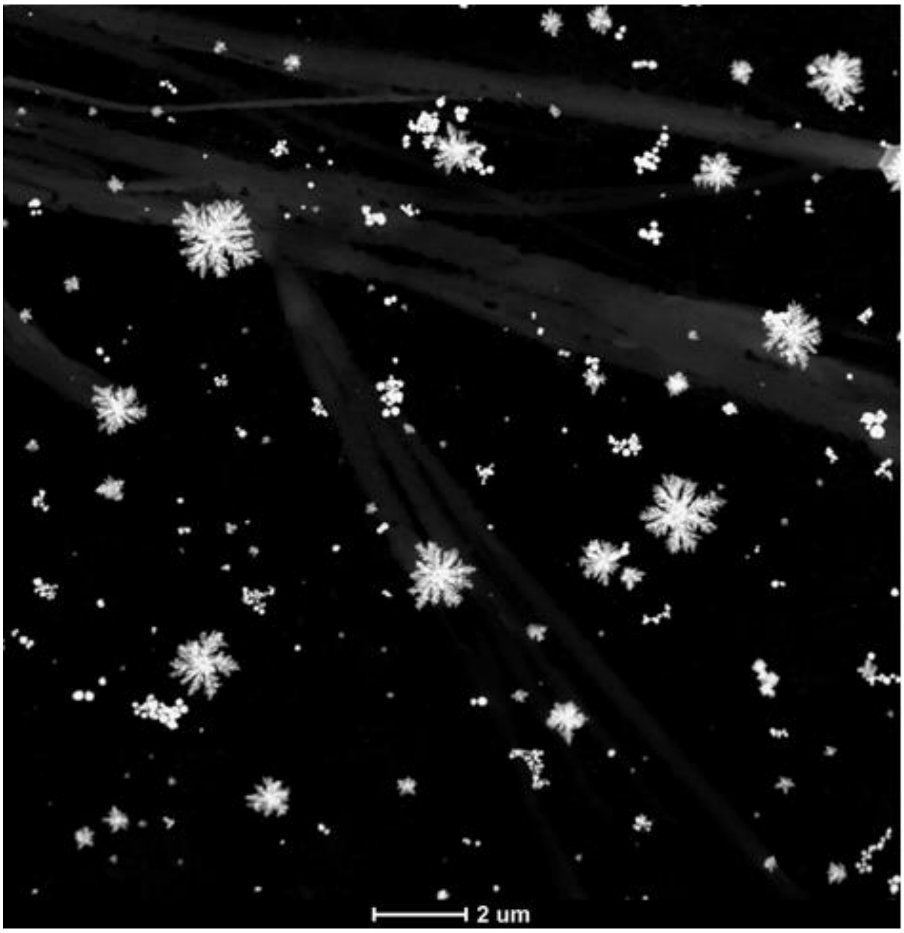
2 um

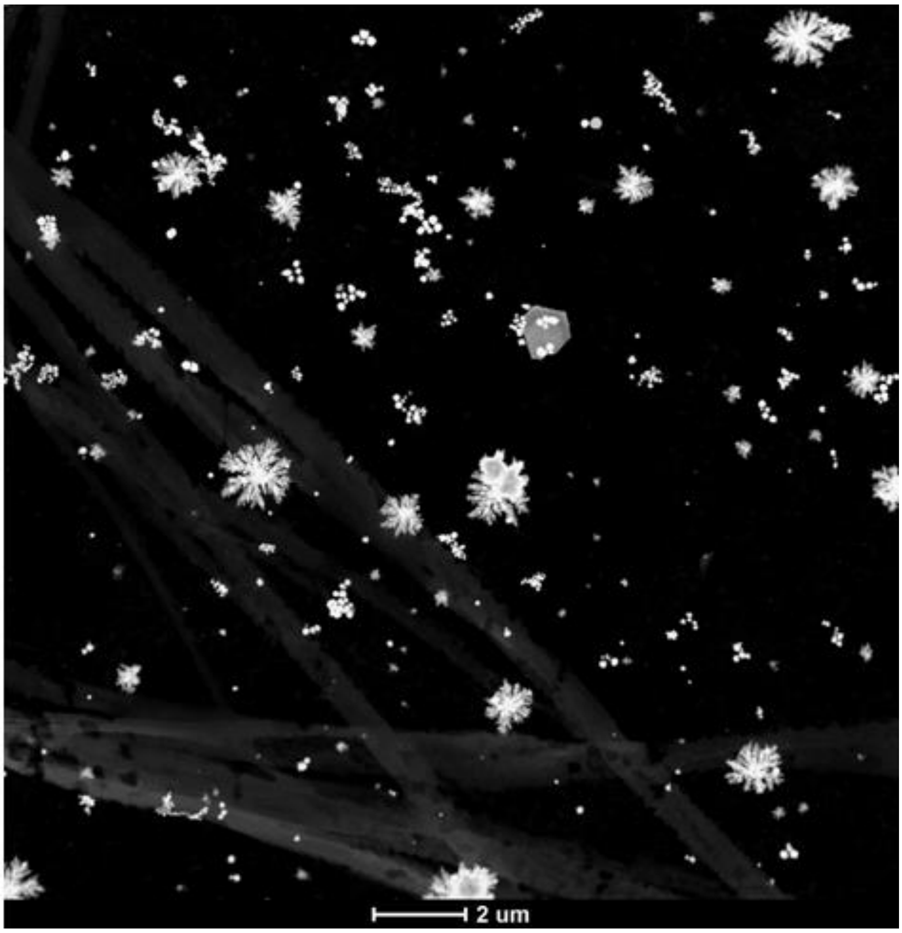
2 um

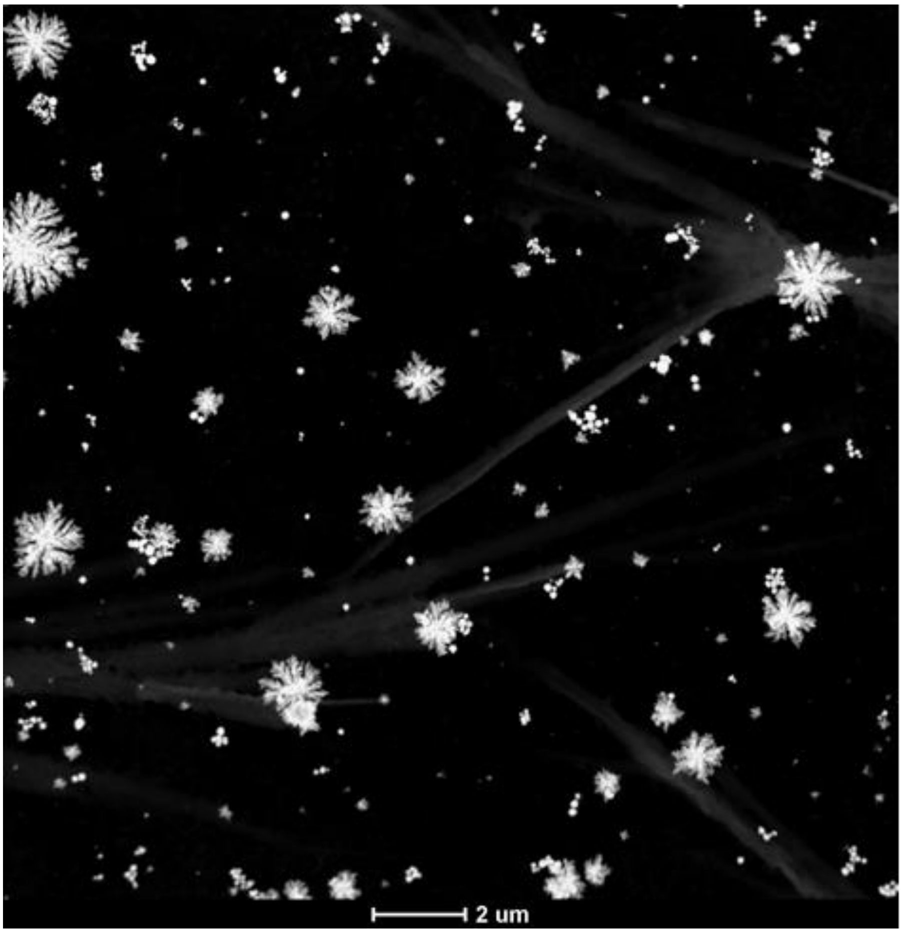
2 um

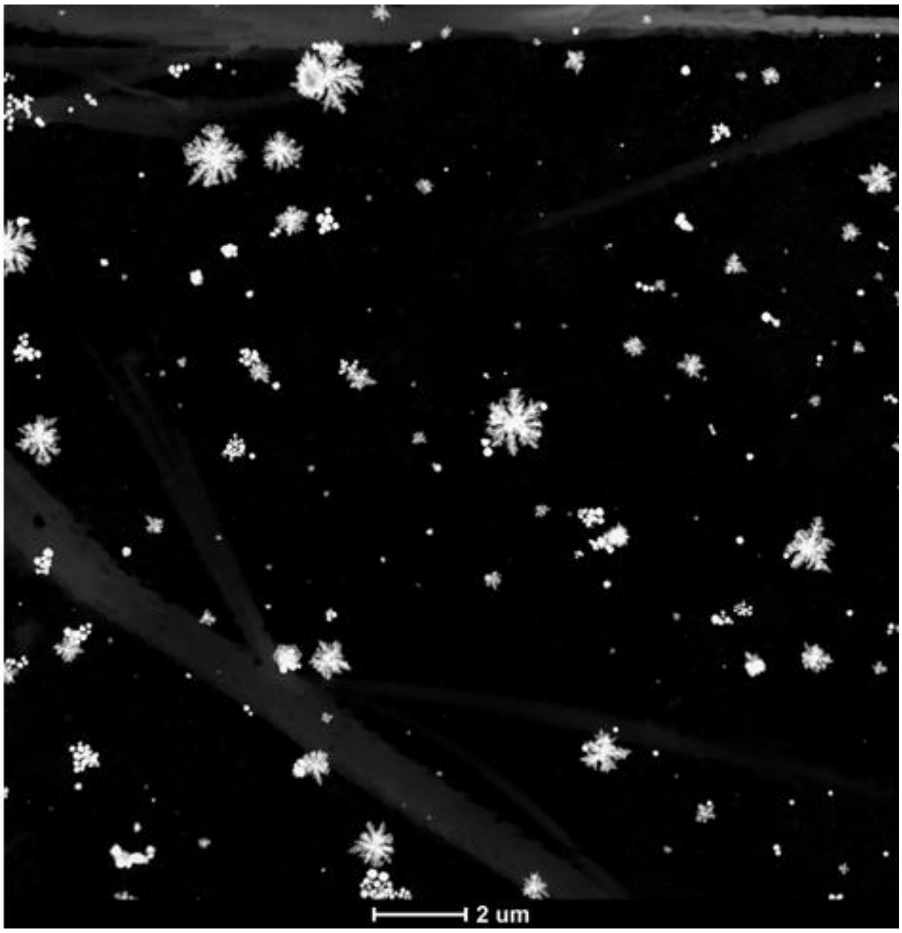
2 um

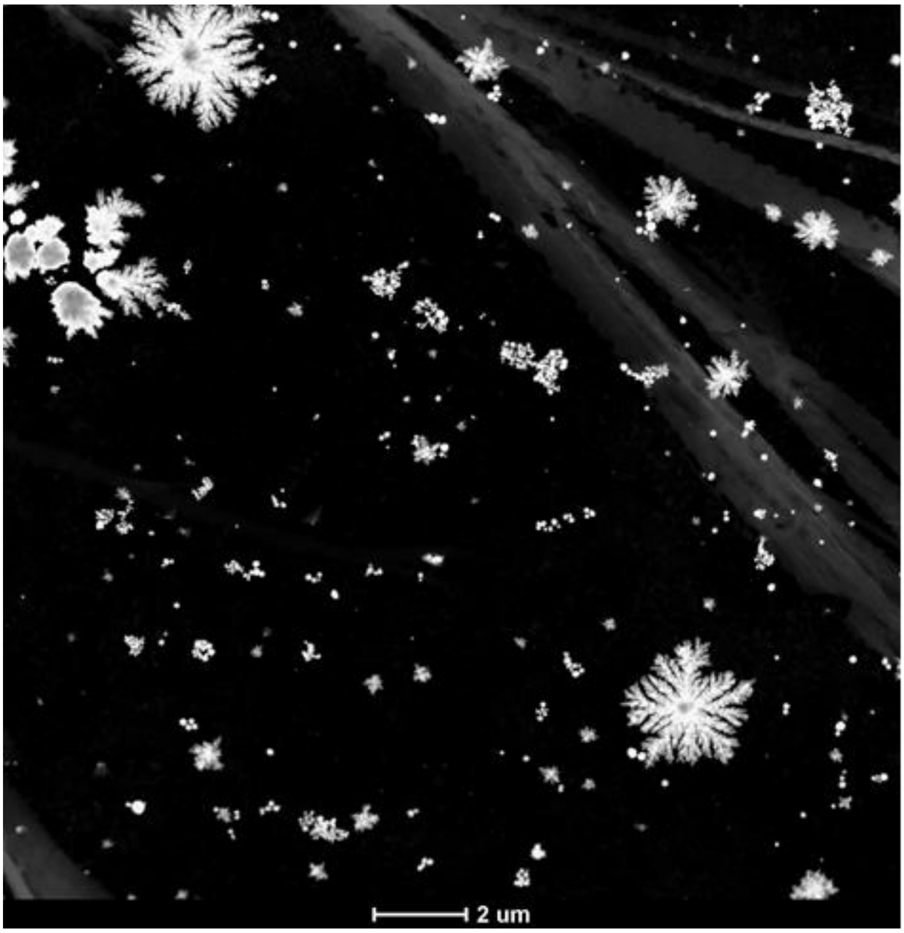
2 um

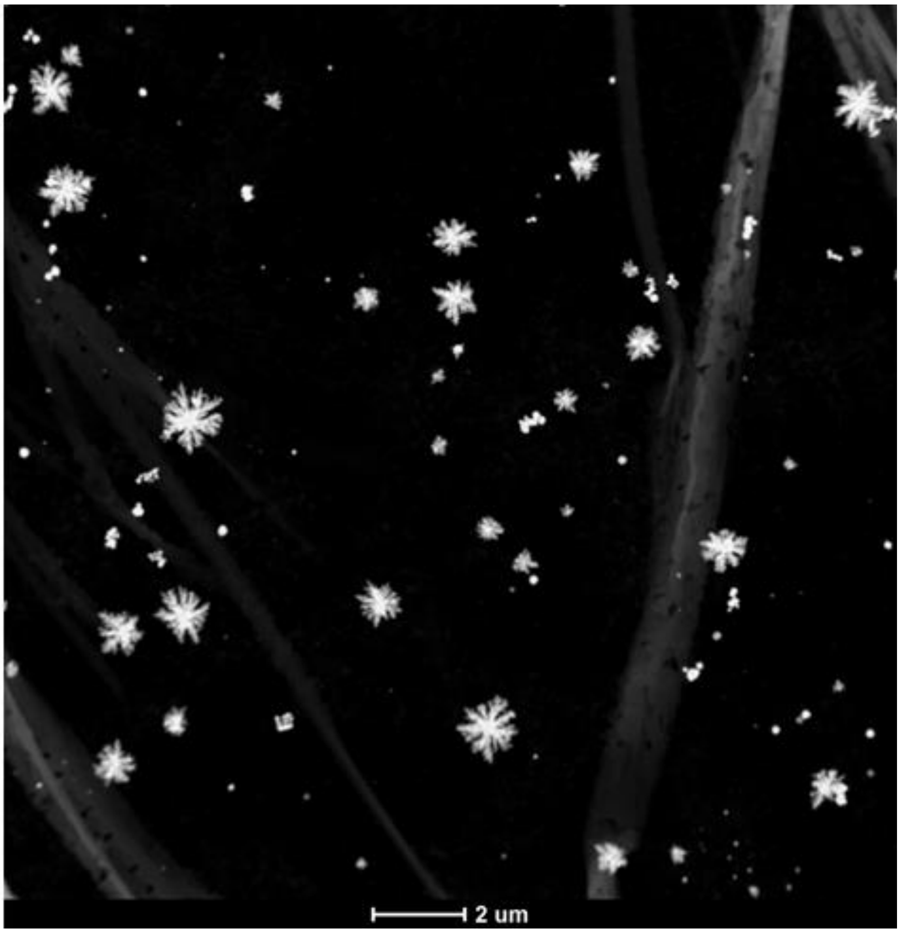

Figure ~~S8~~S4. Six TEM images of the AuNPs in the sample solution collected in phase 1. Size histograms in Figure 2c were from these images. The particles have two different sizes (~950 um and ~170 nm) and shapes (dendritic and spherical). Large rod-shaped structures of weak contrast in the background of the images are due to damages or impurities on the supporting carbon films of the TEM grid.

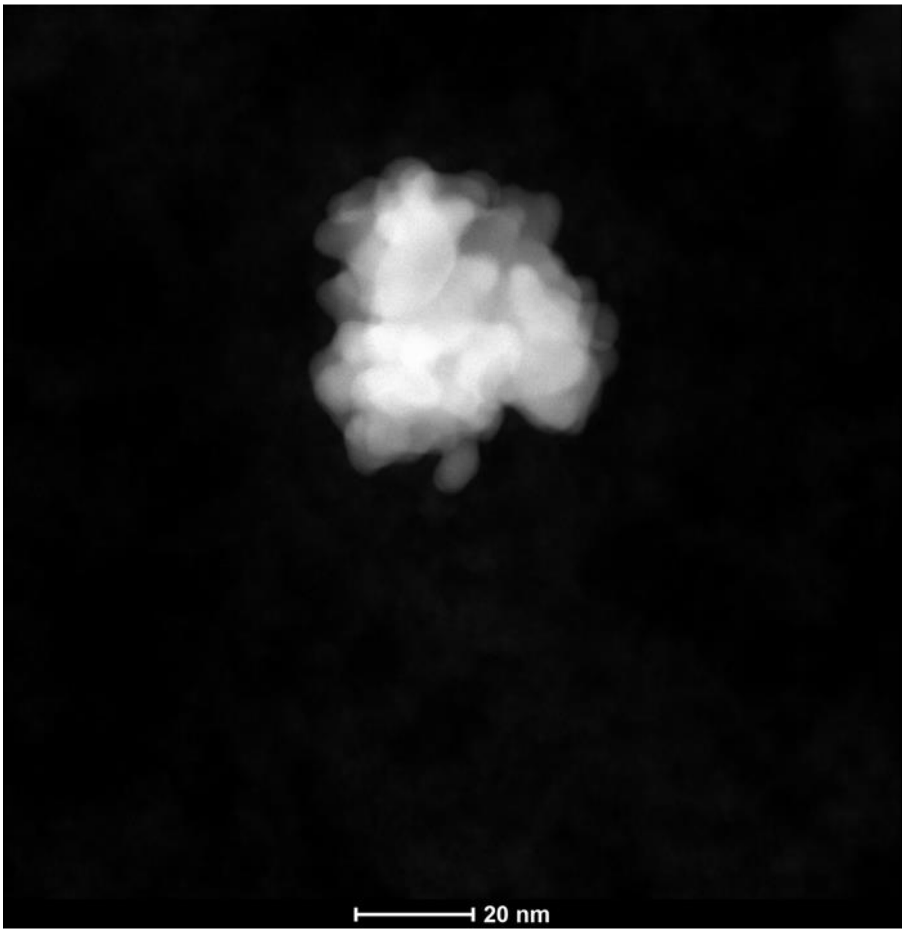

Figure ~~S7~~S5. A magnified TEM image of a raspberry-shaped AuNP. The morphology of the raspberry-shaped AuNPs indicates that such particles would be formed by aggregation of smaller (5~7 nm) AuNPs. Formation of such small AuNPs upon femtosecond laser ablation was reported elsewhere [30-32].

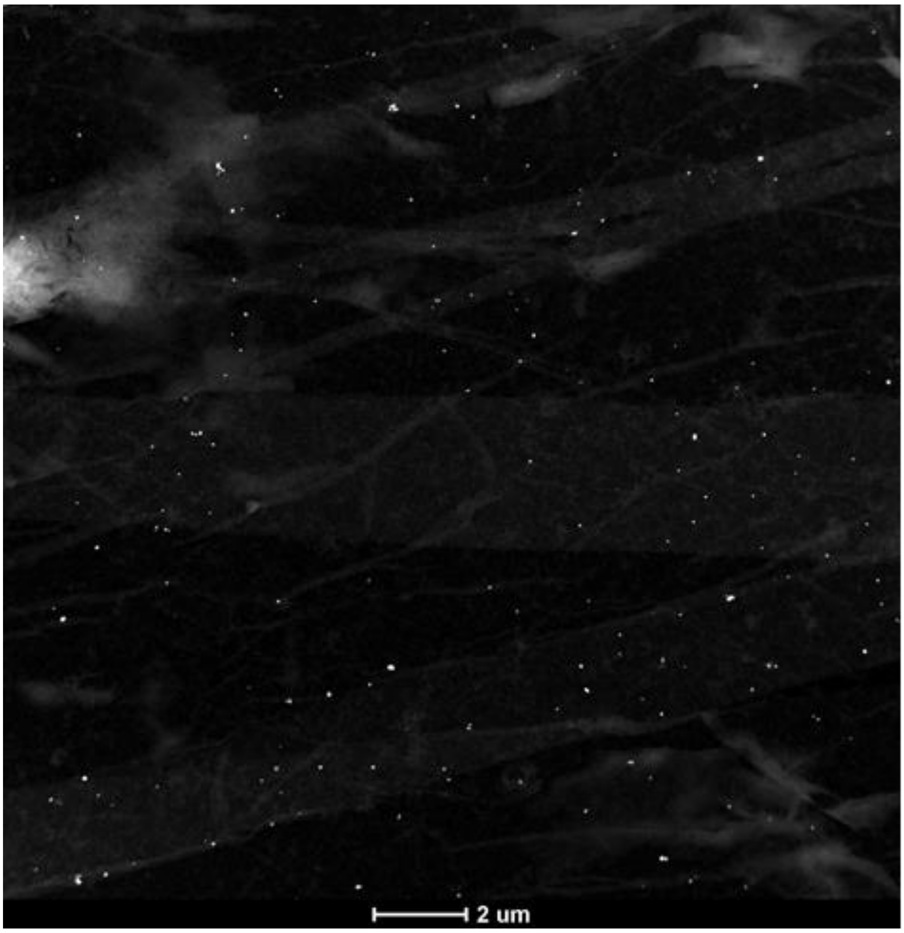
2 um

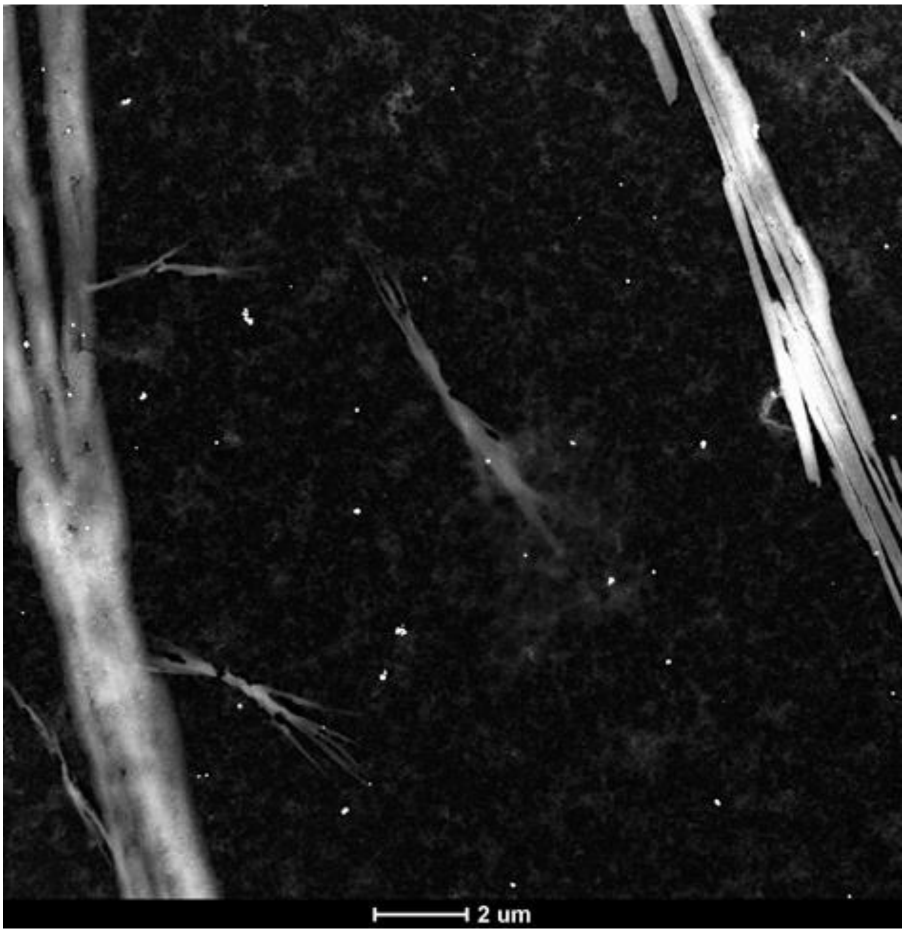
2 um

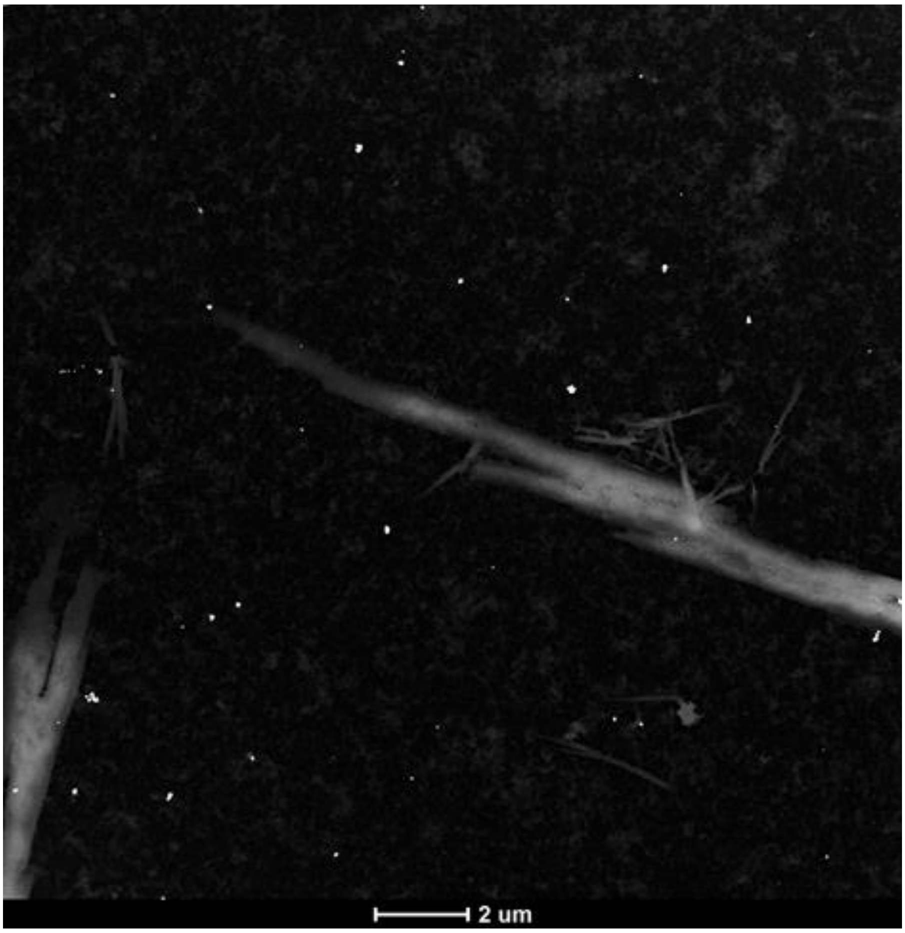
2 um

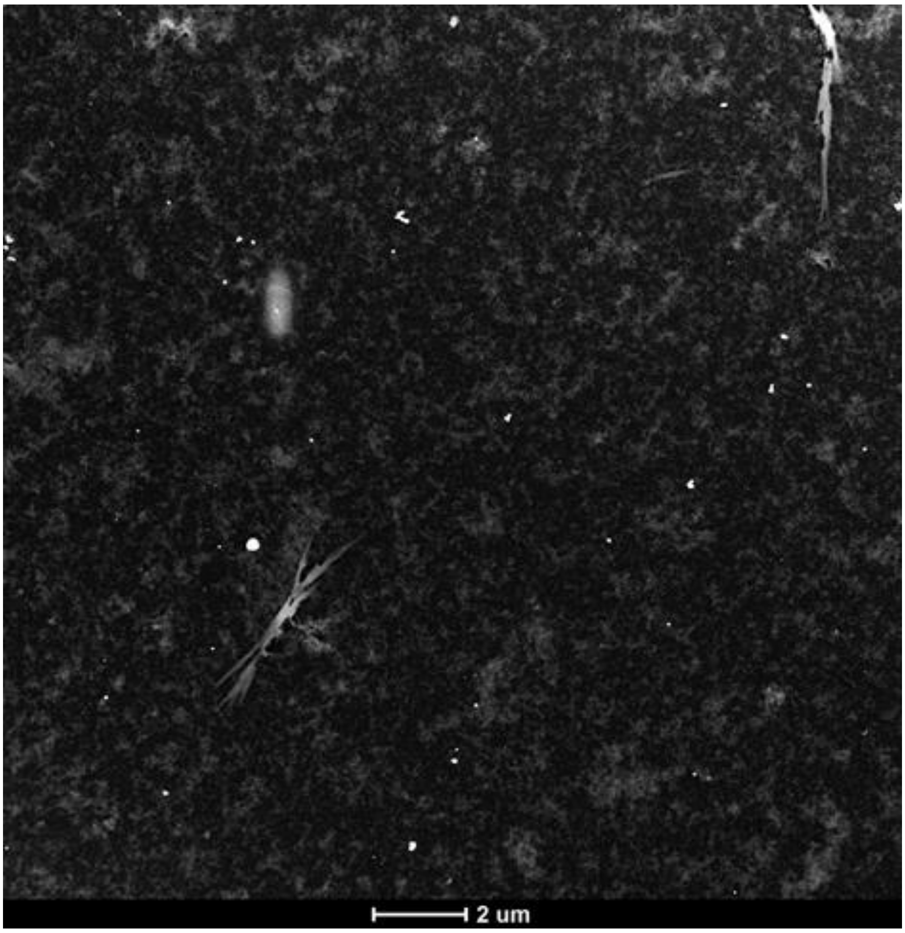
2 um

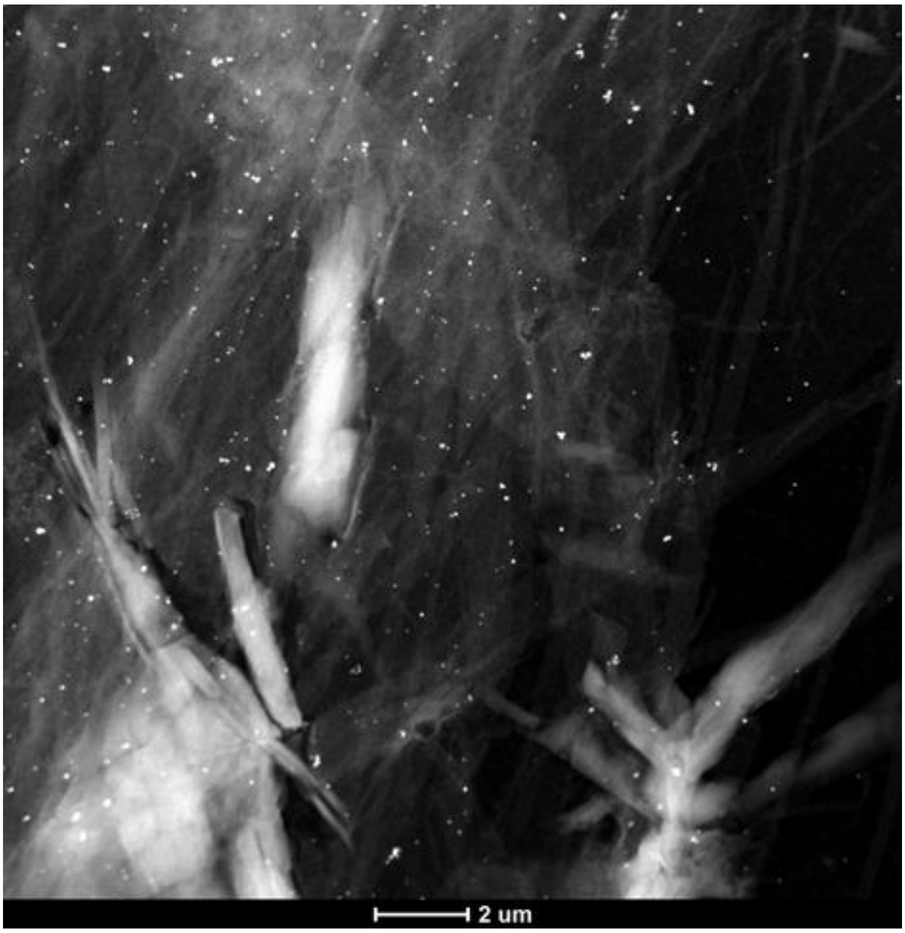
2 um

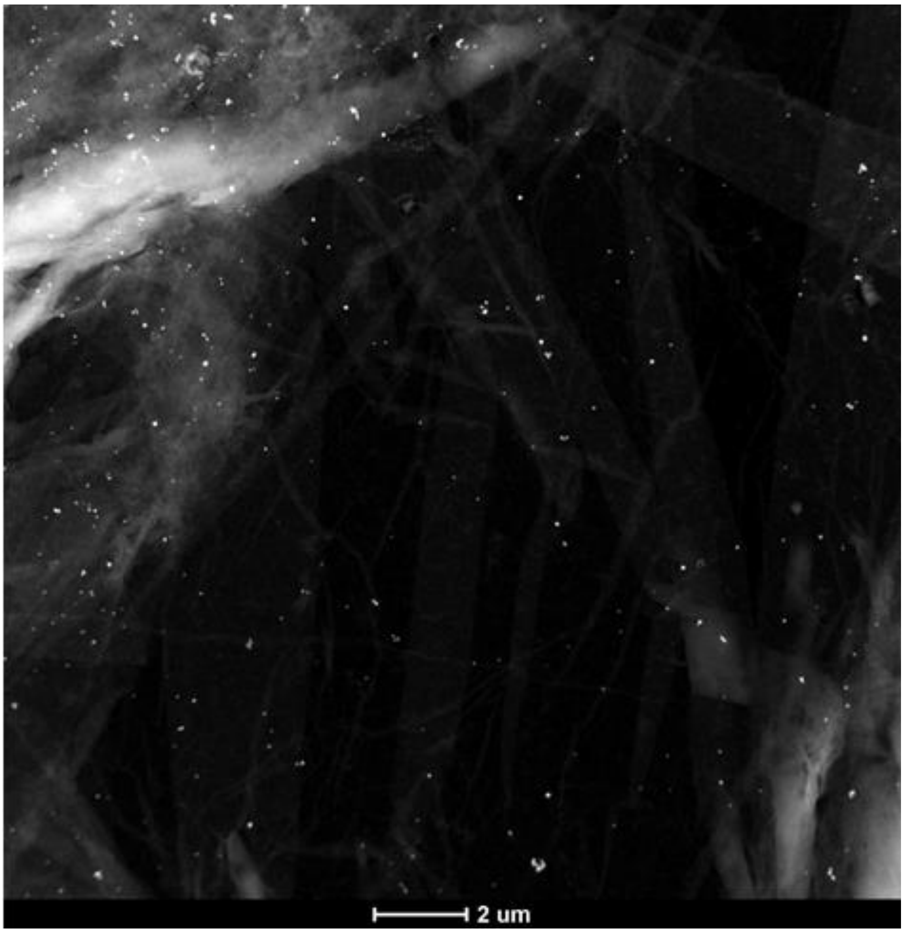

Figure S9S6. Six TEM images of the AuNPs in the sample solution which is collected at phase 2. Size histograms in Figure 2c were from these images. Only the raspberry-shaped particles (~50 nm) are observable. Large rod-shaped structures of weak contrast in the background of the images are due to damages or impurities on the supporting carbon films of the TEM grid.

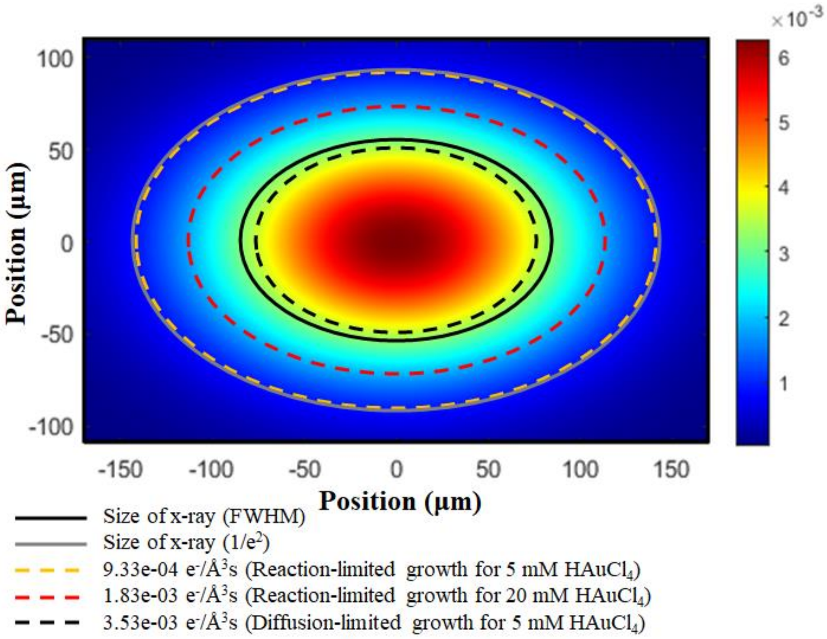

Figure ~~S6~~S7. Spatial distribution of the photoelectrons generated by an x-ray pulse. As shown in Figures 3 and 5 of the main text, we suggest that two different types of AuNPs can be formed under our TRXL experimental condition because AuNPs can be produced via two different growth mechanisms, diffusion-limited and reaction-limited growth. To quantitatively support our suggestion, we estimated the rate of photoelectron generation per unit volume, $\Phi_e(x, y)$, in the liquid jet upon exposure to an x-ray pulse. The resulting distribution of $\Phi_e(x, y)$ is shown as a contour plot. The values are shown in $e^-/(Å^3 \cdot s)$ units. By comparing the $\Phi_e(x, y)$ with the previously reported electron dose rate values for the reaction-limited and diffusion-limited growth of AuNPs [2], we confirmed that the photoelectron density at the central region of the x-ray pulse (especially within the FWHM size of the x-ray pulse) is sufficiently high for AuNPs to grow via diffusion-limited mechanism, whereas the density at the boundary (especially at the outside of the FWHM size of the x-ray pulse) is quite low, allowing the AuNPs grow through a reaction-limited growth mechanism. The detailed procedures for the calculation of

the spatial distribution of the photoelectrons and its comparison to electron dose rates are described in the “Simulation of photoelectron generation in liquid jet subjected to x-ray pulse” section in the SI.

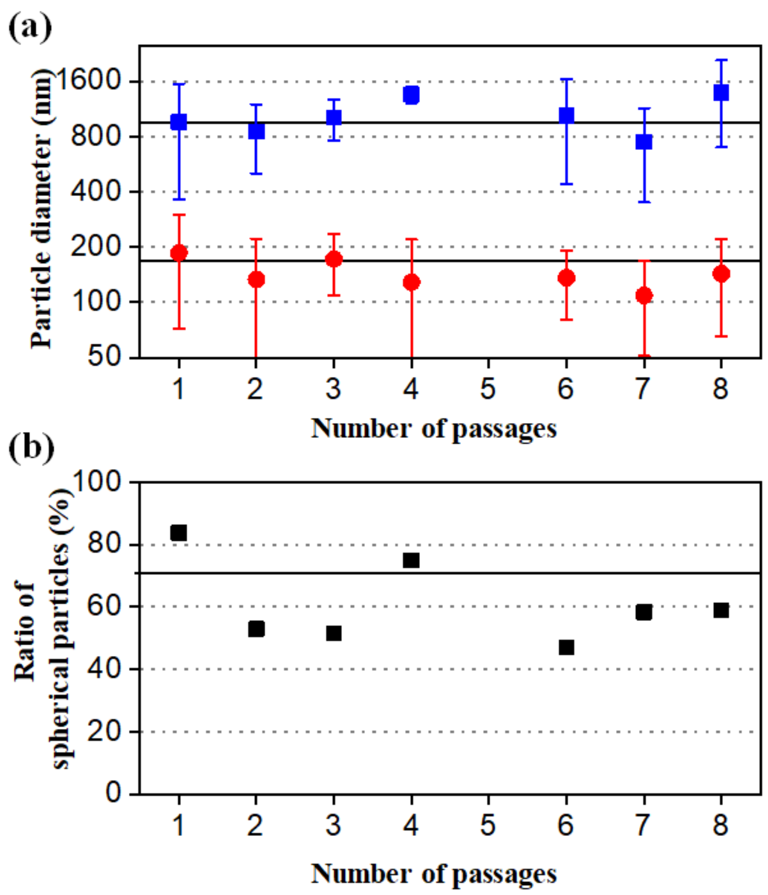

Figure ~~S5~~S8**.** Evolution of the size and the number of dendritic and spherical AuNPs in the sample solution upon exposures to the laser and x-ray pulses. By using TEM measurements, we investigated the size, number, and shape of AuNPs in the samples that passed through the x-ray and laser interaction region from one to eight times. A more detailed procedure is described in the main text. Note that it was not possible to detect the AuNPs in the measured TEM images for the solution exposed to x-ray and laser pulses five times, might due to a failure of sampling, so the data for the solution is missing. (a) The size of each of the dendritic (blue squares) and spherical AuNPs (red circles) as a function of the number of passages to x-ray

and laser pulses. The size of the AuNPs does not show strong dependence on the number of exposures to x-ray and laser pulses. The black solid lines represent the average of the size of each shape of particles. Note that y-axis is on a logarithmic scale. (b) The number ratio of the spherical AuNPs among the total (spherical and dendritic) AuNPs. The ratio does not show strong dependence on the number of exposures to x-ray and laser pulses. The black solid lines represent the average of the ratio of the spherical AuNPs.

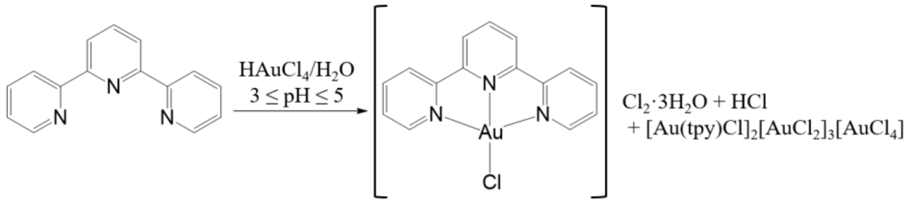

Figure ~~S1~~S9. Synthesis of [Au(tpy)Cl]Cl_2. An equimolar amount of $HAuCl_4$ and terpyridine was refluxed in H_2O at pH 3−5 for 24 h, resulting in the desired [Au(tpy)Cl]Cl_2 in 80% yield along with a trace of $[Au(tpy)Cl]_2[AuCl_2]_3[AuCl_4]$.

Supporting References

1. Berger, M.; Hubbell, J.; Seltzer, S.; Chang, J.; Coursey, J.; Sukumar, R.; Zucker, D.; Olsen, K. *XCOM: Photon Cross Section Database*, (version 3.1); [Online] Available: https://dx.doi.org/10.18434/T48G6X (accessed on 2020/08/24). National Institute of Standards and Technology: Gaithersburg, Maryland, United States, 2010.
2. Zhang, Y.C.; Keller, D.; Rossell, M.D.; Erni, R. Formation of Au Nanoparticles in Liquid Cell Transmission Electron Microscopy: From a Systematic Study to Engineered Nanostructures. *Chem. Mater.* **2017**, *29*, 10518-10525.
3. Parr, R.; Weitao, Y. *Density-Functional Theory of Atoms and Molecules*; Oxford University Press: Oxford, USA, 1994.
4. Bochevarov, A.D.; Harder, E.; Hughes, T.F.; Greenwood, J.R.; Braden, D.A.; Philipp, D.M.; Rinaldo, D.; Halls, M.D.; Zhang, J.; Friesner, R.A. Jaguar: A High-Performance Quantum Chemistry Software Program With Strengths in Life and Materials Sciences. *Int. J. Quantum Chem.* **2013**, *113*, 2110-2142.
5. Tannor, D.J.; Marten, B.; Murphy, R.; Friesner, R.A.; Sitkoff, D.; Nicholls, A.; Ringnalda, M.; Goddard, W.A.; Honig, B. Accurate First Principles Calculation of Molecular Charge-Distributions and Solvation Energies from Ab-Initio Quantum-Mechanics and Continuum Dielectric Theory. *J. Am. Chem. Soc.* **1994**, *116*, 11875-11882.
6. Marten, B.; Kim, K.; Cortis, C.; Friesner, R.A.; Murphy, R.B.; Ringnalda, M.N.; Sitkoff, D.; Honig, B. New Model for Calculation of Solvation Free Energies: Correction of Self-Consistent Reaction Field Continuum Dielectric Theory for Short-Range Hydrogen-Bonding Effects. *J. Phys. Chem.* **1996**, *100*, 11775-11788.
7. Becke, A.D. A New Mixing of Hartree-Fock and Local Density-Functional Theories. *J. Chem. Phys.* **1993**, *98*, 1372-1377.
8. Becke, A.D. Density-Functional Exchange-Energy Approximation with Correct Asymptotic-Behavior. *Phys. Rev. A* **1988**, *38*, 3098-3100.
9. Grimme, S.; Antony, J.; Ehrlich, S.; Krieg, H. A Consistent and Accurate Ab Initio Parametrization of Density Functional Dispersion Correction (DFT-D) for the 94 Elements H-Pu. *J. Chem. Phys.* **2010**, *132*.
10. Hay, P.J.; Wadt, W.R. Ab Initio Effective Core Potentials for Molecular Calculations. Potentials for the Transition Metal Atoms Sc to Hg. *J. Chem. Phys.* **1985**, *82*, 270-283.
11. Hay, P.J.; Wadt, W.R. Ab Initio Effective Core Potentials for Molecular Calculations. Potentials for K to Au Including the Outermost Core Orbitals. *J. Chem. Phys.* **1985**, *82*, 299-310.
12. Wadt, W.R.; Hay, P.J. Ab Initio Effective Core Potentials for Molecular Calculations. Potentials for Main Group Elements Na to Bi. *J. Chem. Phys.* **1985**, *82*, 284-298.
13. Ditchfield, R.; Hehre, W.J.; Pople, J.A. Self-Consistent Molecular-Orbital Methods. IX. An Extended Gaussian-Type Basis for Molecular-Orbital Studies of Organic Molecules. *J. Chem. Phys.* **1971**, *54*, 724-728.
14. Dunning Jr, T.H. Gaussian Basis Sets for Use in Correlated Molecular Calculations. I. The Atoms Boron through Neon and Hydrogen. *J. Chem. Phys.* **1989**, *90*, 1007-1023.
15. Edinger, S.R.; Cortis, C.; Shenkin, P.S.; Friesner, R.A. Solvation Free Energies of Peptides: Comparison of Approximate Continuum Solvation Models With Accurate Solution of the Poisson−Boltzmann Equation. *J. Phys. Chem. B* **1997**, *101*, 1190-1197.
16. Friedrichs, M.; Zhou, R.; Edinger, S.R.; Friesner, R.A. Poisson− Boltzmann Analytical Gradients for Molecular Modeling Calculations. *J. Phys. Chem. B* **1999**, *103*, 3057-3061.

17. Isse, A.A.; Gennaro, A. Absolute Potential of the Standard Hydrogen Electrode and the Problem of Interconversion of Potentials in Different Solvents. *J. Phys. Chem. B* **2010**, *114*, 7894-7899.
18. Kennedy, I.; Geering, H.; Rose, M.; Crossan, A. A Simple Method to Estimate Entropy and Free Energy of Atmospheric Gases From Their Action. *Entropy* **2019**, *21*, 454.
19. Bartels, D.M.; Takahashi, K.; Cline, J.A.; Marin, T.W.; Jonah, C.D. Pulse Radiolysis of Supercritical Water. 3. Spectrum and Thermodynamics of the Hydrated Electron. *J. Phys. Chem. A* **2005**, *109*, 1299-1307.
20. Kumar, A.; Walker, J.A.; Bartels, D.M.; Sevilla, M.D. A Simple Ab Initio Model for the Hydrated Electron That Matches Experiment. *J. Phys. Chem. A* **2015**, *119*, 9148-9159.
21. Shiraishi, H.; Sunaryo, G.R.; Ishigure, K. Temperature Dependence of Equilibrium and Rate Constants of Reactions Inducing Conversion Between Hydrated Electron and Atomic Hydrogen. *J. Phys. Chem.* **1994**, *98*, 5164-5173.
22. Ben-Naim, A.Y. *Solvation Thermodynamics*; Springer Science & Business Media: 1987.
23. Pollard, T.P.; Beck, T.L. The Thermodynamics of Proton Hydration and the Electrochemical Surface Potential of Water. *J. Chem. Phys.* **2014**, *141*, 18C512.
24. Henglein, A.; Meisel, D. Radiolytic Control of the Size of Colloidal Gold Nanoparticles. *Langmuir* **1998**, *14*, 7392-7396.
25. Henglein, A. Reduction of $Ag(CN)_2^-$ on Silver and Platinum Colloidal Nanoparticles. *Langmuir* **2001**, *17*, 2329-2333.
26. Mosseri, S.; Henglein, A.; Janata, E. Reduction of Dicyanoaurate (I) in Aqueous Solution: Formation of Nonmetallic Clusters and Colloidal Gold. *J. Phys. Chem.* **1989**, *93*, 6791-6795.
27. Arblaster, J. Thermodynamic Properties of Gold. *J. Phase Equilib. Diff.* **2016**, *37*, 229-245.
28. Vanýsek, P. Electrochemical Series. In *CRC Handbook of Chemistry and Physics*, 85th ed.; Lide, D.R., Ed. CRC press: Boca Raton, Florida, 2004; Vol. 85, p. 8-23.
29. Messori, L.; Abbate, F.; Marcon, G.; Orioli, P.; Fontani, M.; Mini, E.; Mazzei, T.; Carotti, S.; O'Connell, T.; Zanello, P. Gold (III) Complexes as Potential Antitumor Agents: Solution Chemistry and Cytotoxic Properties of Some Selected Gold (III) Compounds. *J. Med. Chem.* **2000**, *43*, 3541-3548.
30. Plech, A.; Kotaidis, V.; Lorenc, M.; Boneberg, J. Femtosecond Laser Near-Field Ablation From Gold Nanoparticles. *Nat. Phys.* **2006**, *2*, 44-47.
31. Werner, D.; Furube, A.; Okamoto, T.; Hashimoto, S. Femtosecond Laser-Induced Size Reduction of Aqueous Gold Nanoparticles: In Situ and Pump−Probe Spectroscopy Investigations Revealing Coulomb Explosion. *J. Phys. Chem. C* **2011**, *115*, 8503-8512.
32. González-Rubio, G.; Guerrero-Martínez, A.s.; Liz-Marzán, L.M. Reshaping, Fragmentation, and Assembly of Gold Nanoparticles Assisted by Pulse Lasers. *Acc. Chem. Res.* **2016**, *49*, 678-686.
